# Mendelian randomization study on insulin resistance and risk of hypertension and cardiovascular disease

**DOI:** 10.1038/s41598-023-46983-3

**Published:** 2024-03-14

**Authors:** Fangfang Zhang, Zhimin Yu

**Affiliations:** 1 Department of Outpatient, Taihe Hospital, Hubei University of Medicine, Shiyan, 442000 Hubei China; 2 Department of Geriatrics, Taihe Hospital, Hubei University of Medicine, Shiyan, 442000 China

**Keywords:** Cardiovascular diseases, Arrhythmias, Heart failure, Hypertension, Vascular diseases, Endocrinology, Risk factors

## Abstract

Observational studies have suggested that insulin resistance (IR) is associated with hypertension and various cardiovascular diseases. However, the presence of a causal relationship between IR and cardiovascular disease remains unclear. Here, we applied Mendelian randomization (MR) approaches to address the causal association between genetically determined IR and the risk of cardiovascular diseases. Our primary genetic instruments comprised 53 SNPs associated with IR phenotype from a GWAS of up to 188,577 participants. Genetic association estimates for hypertension and venous thromboembolism (VTE) were extracted from UK Biobank, estimates for atrial fibrillation (AF) were extracted from the hitherto largest GWAS meta-analysis on AF, estimates for heart failure were extracted from HERMES Consortium, estimates for peripheral artery disease (PAD) and aortic aneurysm were extracted from the FinnGen Study. The main analyses were performed using the random-effects inverse-variance weighted approach, and complemented by sensitivity analyses and multivariable MR analyses. Corresponding to 55% higher fasting insulin adjusted for body mass index, 0.46 mmol/L lower high-density lipoprotein cholesterol and 0.89 mmol/L higher triglyceride, one standard deviation change in genetically predicted IR was associated with increased risk of hypertension (odds ratio (OR) 1.06, 95% CI 1.04–1.08; *P* = 1.91 × 10^–11^) and PAD (OR 1.90, 95% CI 1.43–2.54; *P* = 1.19 × 10^–5^). Suggestive evidence was obtained for an association between IR and heart failure (OR per SD change in IR: 1.19, 95% CI 1.01–1.41, *P* = 0.041). There was no MR evidence for an association between genetically predicted IR and atrial fibrillation, VTE, and aortic aneurysm. Results were widely consistent across all sensitivity analyses. In multivariable MR, the association between IR and PAD was attenuated after adjustment for lipids (*P* = 0.347) or BMI (*P* = 0.163). Our findings support that genetically determined IR increases the risk of hypertension and PAD.

## Introduction

Insulin resistance (IR), a systemic disorder characterized by decreased sensitivity to insulin with an impaired ability to maintain normal glucose metabolism, has been implicated in various metabolic disorders^1,2^. A number of prospective observational studies have indicated that IR is related to the risk of hypertension^3^ and various other cardiovascular diseases (CVDs), including atrial fibrillation^4^, heart failure^5^, peripheral artery disease (PAD)^6^, and venous thromboembolism (VTE)^7^. However, the associations are inconsistent regarding IR with risk of atrial fibrillation^4,8^ and heart failure^5,9–11^; whereas data on causal role of IR for hypertension^3^, PAD^6^, VTE^12^, and aortic aneurysm are scarce. Moreover, given that most of the available data on IR and CVDs originates from observational studies, which are susceptible to confounding and reverse causality bias, the causal nature of relationships between IR and various CVDs remains to be elucidated.

Mendelian randomization (MR) is an approach that utilizes genetic variants as instruments for an exposure of interest to determine whether the exposure is a cause of a disease outcome^13^. As genetic variants are randomly assigned at meiosis and fixed after conception, MR limits the effect of confounding and reverse causation in the study of an association^14^. The MR design has been previously applied to investigate the association of IR with certain CVDs, including coronary artery disease, myocardial infarction, and ischemic stroke^15^. However, the associations of IR with many other CVDs, including hypertension, atrial fibrillation, heart failure, PAD, VTE, and aortic aneurysm, have not yet been investigated using this approach. We therefore conducted a MR study to examine the causal relationships between IR and these 6 CVD outcomes.

## Methods

### Study design

**Figure 1:**
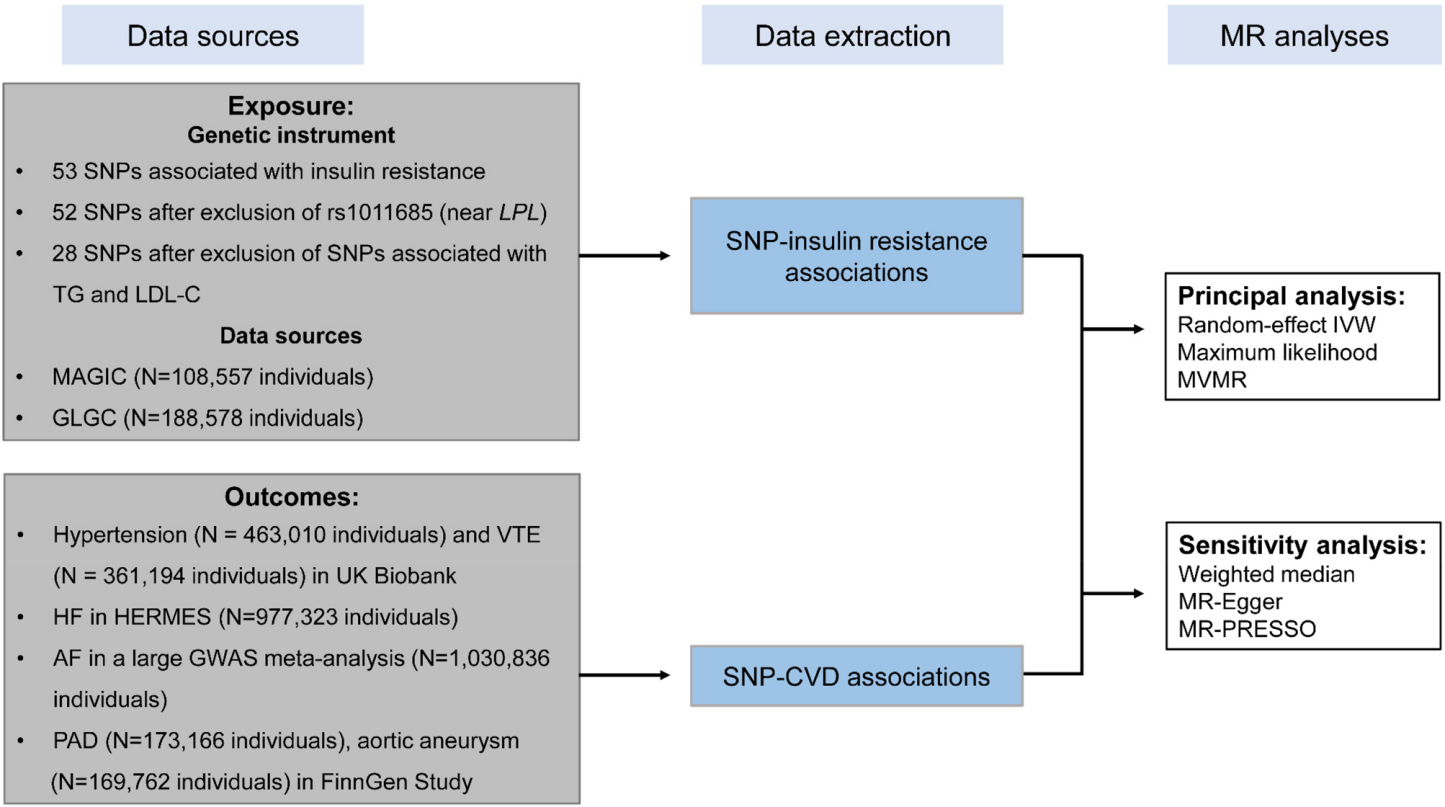
Schematic representation for data sources and methods applied in Mendelian randomization study (Microsoft PowerPoint, version 2111, URL: https://www.office.com/).

We applied a two-sample MR design to estimate whether IR is causally associated with hypertension, atrial fibrillation, heart failure, PAD, VTE, and aortic aneurysm. MR relies on 3 principle assumptions to provide unbiased estimates^16^: (1) Instrument variants should be strongly related to exposure; (2) Instrument variants should be independent of any confounders of the exposure-outcome association; (3) Instrument variants exert impact on outcomes entirely through the exposure. In this MR study, genetic instruments proxying IR (exposure) were derived from the largest publicly available Genome-Wide Association Study (GWAS)^17^. Publicly available summary-level data for 6 outcomes were extracted from UK Biobank^18^, a large GWAS meta-analysis on AF^19^, HERMES Consortium (Heart Failure Molecular Epidemiology for Therapeutic Targets Consortium)^20^, and the FinnGen Study^21^ (depicted in Fig. 1). Ethics consents were obtained from participants of all the aforementioned GWASs. No individual-level data were utilized in this MR study.

### Genetic instruments selection

Genetic instruments for IR were derived from a meta-analyzed GWAS based on the MAGIC (Meta-Analyses of Glucose- and Insulin-related traits Consortium)^22^ and GLGC (Global Lipids Genetics Consortium)^23^, comprising up to 188,577 participants of European-descent^17^. In that study, fasting insulin data adjusted for body mass index (BMI), high-density lipoprotein cholesterol (HDL-C), and triglyceride (TG) from individual GWAS were integrated to uncover loci related to a phenotypic pattern indicative of IR^17^. A total of 53 lead single nucleotide polymorphisms (SNPs) that were associated with IR phenotype at genome-wide significance level (*P* < 3.1 × 10^–08^) were identified as genetic instruments^17^. These 53 SNPs had been verified to be strongly associated with gold-standard measures of insulin sensitivity in several independent cohorts, either using euglycemic clamp or insulin suppression test (*P* = 4.3 × 10^–6^), or using a frequently sampled oral glucose tolerance test (*P* = 7.3 × 10^–10^)^17^.

Since the beta and SE values for the association of SNP with IR phenotype were not available in that GWAS study, we obtained the SNP-exposure estimates from a meta-analysis of the MAGIC and GLGC consortium performed by Wang et al.^24^. Based on MAGIC and GLGC study, the investigators recalculated the beta and SE values for these 53 SNP associations with IR phenotype using a fixed-effect inverse-variance weighted approach^24^. SNPs were then linkage disequilibrium-pruned (*r*^2^ < 0.01, distance threshold = 5000 kb) to identify independent instruments. All SNPs used as genetic instruments were available in the outcome GWASs.

Our MR results express results in the outcome per 1 SD change in the exposure. 1 SD change of IR phenotype corresponds to 55% higher fasting insulin adjusted for BMI, 0.46 mmol/L lower HDL-C and 0.89 mmol/L higher TG, indicating lower insulin sensitivity^17^. The reported odds ratios (ORs) in our study represent 1 SD change in the IR phenotype.

### Data sources for outcomes

We extracted summary statistics for IR-associated SNPs with hypertension (54,358 cases and 408,652 controls) and VTE (4620 cases and 356,574 controls) from the Neale Lab UK Biobank GWAS^18^, a cohort study of approximately 500,000 individuals from UK general population between 2006 and 2010. In UK Biobank, the primary hypertension was defined using *international classification of diseases 10* diagnosis code I10 (*ICD-10*: I10) from discharge registries. VTE was defined based on self-reported at baseline (internal UK Biobank codes 1068, 1093, and 1094) or subsequently diagnosis of hospital episodes (*ICD‐9* 415.1, 416.2, 451–453 and *ICD‐10* I26, I80–I82) or causes of death (*ICD‐10* I26 and I80-I82), including both incident and prevalent VTE individuals. The data for hypertension and VTE were accessed through MR-Base platform with ukb-b-12493 and ukb-d-I9_VTE, respectively^25^.

Summary statistics for IR-associated SNPs with atrial fibrillation were derived from a GWAS meta-analysis which included 60,620 atrial fibrillation cases and 970,216 controls^19^. Summary statistics for IR-associated SNPs with heart failure were derived from the GWAS of Heart Failure Molecular Epidemiology for Therapeutic Targets (HERMES) Consortium, which included 47,309 heart failure cases and 930,014 controls^20^. Summary statistics for the IR-associated SNPs with PAD (5323 cases and 167,843 controls) and aortic aneurysm (1919 cases and 167,843 controls) were extracted from the latest data release from the FinnGen Study (Release 4)^21^.

The definitions of the six CVD outcomes are detailed in Supplementary Table S1. Supplementary Table S2 displayed detailed information on data sources. All participants included in this MR study were of European ancestry. There was no participant overlap between GWAS of exposure and CVD outcome except for atrial fibrillation, where only limited overlap (1.6%) was found.

### Statistical analysis

In primary analyses, we utilized the random-effects inverse-variance weighted (IVW) method to estimate the causal associations of instrument variables for exposures with CVD outcomes^26^. The Wald ratio of the SNP-outcome association and the SNP-exposure association was treated as the causal estimate for each SNP. Then these estimates were pooled using random-effects model. This method provides reliable MR estimate when three principal MR assumptions are valid. We measured the heterogeneity between individual SNPs with the Cochran’s Q statistic, *P* < 0.05 indicated horizontal pleiotropy^27^.

In the secondary analyses, maximum likelihood method was applied to estimate the causal effect^28^. Based on linear model and assumptions of no horizontal pleiotropy or heterogeneity, this method provides the causal effect through the direct maximization of the likelihood given the SNP-exposure and SNP-outcome effects.

To test the validity and robustness of the causal inferences, we conducted sensitivity analyses using the weighted median^29^, MR-Egger method^30^, and the MR pleiotropy residual sum and outlier test (MR-PRESSO)^31^. The weighted median method gives reliable estimates if over 50% of the weight in the analysis has been derived from valid genetic instruments^29^. Potential directional pleiotropy was assessed with MR-Egger method, where the intercept term constrained to zero suggested the absence of directional pleiotropy^30^. Furthermore, the MR-Egger regression method renders pleiotropy-corrected causal effect estimates though has less precision compared to the weighted median method. MR-PRESSO approach was used to filter outlier instrument variables that are potentially horizontally pleiotropic. Similar to the MR-Egger method, this approach provides outlier SNPs omitted causal estimates^31^.

In subsequent analyses, 52-SNPs, 44-SNPs and 28-SNPs instruments besides the 53-SNPs (all loci) were used. One out of the 53 SNPs, rs1011685 (near *LPL* locus), indicated an inconsistent correlation across phenotypes of IR^24^, was excluded to obtain the 52-SNPs instrument. Further sensitivity analysis was conducted after excluding 9 SNPs that were nominally (*P* < 0.001) associated with BMI^32^(44-SNPs). Of the 53 identified SNPs, 25 loci that previously implicated in TGs or HDL-C level at the genome-wide significance level (*P* < 5 × 10^−8^) were excluded, leaving 28 SNPs for further analysis (28-SNPs, Supplemental Tables S3–S6).

In addition, we performed multivariable MR to dissect effects on the CVD outcomes of each component of the mixed IR phenotype^33^. Three components of IR phenotype, i.e., fasting insulin adjusted for BMI, HDL-C, and TG were included in the multivariable weighted linear regression model. In case a potential or significant causal association was obtained in the main analysis, we conducted multivariable MR to assess whether the association was potentially mediated by lipid phenotypes^23^ or BMI^34^. To this end, we extracted absolute value of the standardized beta coefficient for each of the SNP associations with the individual components of the IR phenotype from MAGIC^22^ and GLGC Consortium^23^. Then fixed-effect IVW approach was employed to meta-analyze these estimates to generate summary-level statistics for the required amount of SNPs.

Effect sizes were reported as ORs with 95% confidence intervals (95% CIs). All tests were two sided and Bonferroni-corrected threshold of *P* < 8.33 × 10^–3^ (0.05 divided by 6 CVD outcomes) was set as the significance level. P values between 8.33 × 10^–3^ and 0.05 were considered as potential evidence of association. The statistical analysis was carried out using RStudio (version 3.6.1) with packages TwoSampleMR, MR pleiotropy residual sum and outlier, and MendelianRandomization. Schematic diagram was created with PowerPoint 2016 (Microsoft Corporation, Redmond, CA) and forest plots were created by using STATA v.13.1MP (College Station, TX, USA). This paper has been reported according to the recently published Strengthening the Reporting of Observational Studies in Epidemiology-Mendelian randomization guidelines^35^. The study protocol and details were not pre-registered.

All methods were carried out in accordance with relevant guidelines and regulations.

## Results

### Genetically determined IR With CVD: principal results

**Figure 2:**
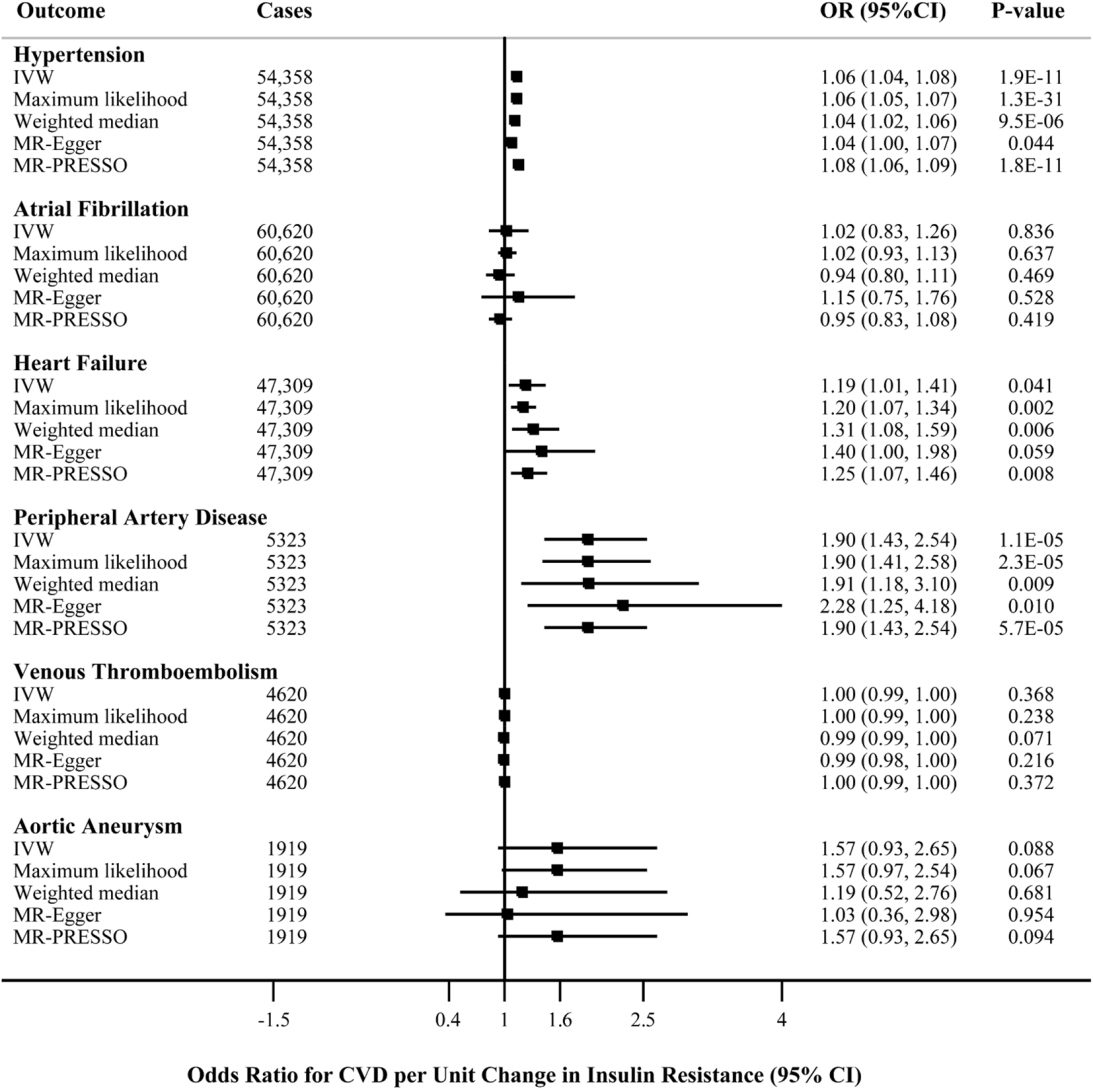
Causal associations of genetically predicted IR with hypertension, atrial fibrillation, and other CVDs based on 53-SNPs instrument. ORs are per 1 SD change in IR exposure (STATA, version 13.1, URL: https://www.stata.com). *CI* confidence interval, *CVD* cardiovascular disease, *IVW* inverse-variance weighted, *IR* insulin resistance, *MR-PRESSO* MR pleiotropy residual sum and outlier test, *OR* odds ratio.

The principal results of genetically predicted IR with CVDs based on 53-SNPs (all loci), 52-SNPs (after excluding rs1011685), and 28-SNPs (after excluding SNPs associated with lipid traits) are reported in Fig. 2, and Supplemental Figs. S2–S3, respectively. The effect estimates for genetic instruments with IR phenotype and with CVDs are displayed in Supplemental Table S7. Genetically instrumented IR was associated with higher risk of hypertension (OR 1.06, 95% CI 1.04–1.08; *P* = 1.91 × 10^–11^) and PAD (OR 1.90, 95% CI 1.43–2.54; *P* = 1.19 × 10^–5^) at the Bonferroni-adjusted level of significance (*P* < 8.33 × 10^–3^) when using 53-SNPs instrument (Fig. 2, Supplemental Fig. S1). The association for hypertension remained statistically significant using either the 52-SNPs or 28-SNPs instrument; potential evidence of association remained for PAD when using the 28-SNPs instrument (Supplemental Figs. S2–S3).

There was potential evidence of association between genetically predicted IR and risk of heart failure (OR 1.19, 95% CI 1.01–1.41, *p* = 0.041). However, this association did not persist after exclusion of rs1011685 in the *LPL* gene region (52-SNPs, OR 1.15, 95% CI 0.94–1.39; *P* = 0.771), or exclusion of SNPs that were significantly associated with TGs or HDL-C (28-SNPs, OR 1.16, 95% CI 0.85–1.59; *P* = 0.356). No significant associations were observed for genetically predicted IR with atrial fibrillation (OR 1.02, 95% CI 0.83–1.26; *P* = 0.836), VTE (OR 1.00, 95% CI 0.99–1.00; *P* = 0.368), or aortic aneurysm (OR 1.57, 95% CI 0.93–2.65; *P* = 0.088) using the 53-SNPs instrument. Similar null associations of genetically predicted IR with atrial fibrillation, VTE and aortic aneurysm were obtained with 52-SNPs or 28-SNPs instruments (Supplemental Figs. S2–S3).

Further analysis was conducted after excluding 9 SNPs that were nominally implicated in BMI (leaving 44-SNPs). We set the threshold at 0.05 to account for potentially pleiotropic effects of IR-associated SNPs. After excluding SNPs that were associated with BMI, MR analyses provided similar estimates to the principal MR analyses including all SNPs (Supplementary Table S8).

### Sensitivity analysis

For most CVD outcomes in MR analysis, significant heterogeneity among SNPs was observed as measured by Cochran Q. We therefore applied random effect models to estimate the effect sizes. However, the outcomes were unlikely influenced by the heterogeneity, as the weighted median approach achieved same direction outcomes compared to the IVW and maximum likelihood method (Supplemental Table S9).

The MR-Egger analysis for hypertension suggested potential directional pleiotropy when using 53-SNP instrument (*P* for intercept = 0.043). However, suggestive evidence for causal association between IR and hypertension remained in pleiotropy-corrected MR-Egger analysis (OR 1.04, 95% CI 1.00–1.07; *P* = 0.043), and weighted median analysis to inspect potential pleiotropy yielded consistent result (OR 1.04, 95% CI 1.02–1.06; *P* = 9.50 × 10^–6^). Meanwhile, after excluding outlier SNPs in MR-PRESSO analysis, the corrected estimate still showed similar effect size to IVW and maximum likelihood-based result (OR 1.08, 95% CI 1.06–1.09; *P* = 1.83 × 10^–11^), establishing the casual relationship between IR and increased hypertension risk. No obvious evidence of directional pleiotropy for other CVD outcomes was observed whenever using the 53-SNPs, 52-SNPs, 44-SNPs, or 28-SNPs instruments, as the intercept values in MR-Egger regression were all close to zero (*P* for intercept all > 0.05, Supplemental Table S9).

Although the IVW analysis suggested a potential association between genetically predicted IR and higher odds of heart failure (OR 1.19, 95% CI 1.01–1.41), further analyses using the maximum likelihood (OR 1.20, 95% CI 1.07–1.34, *P* = 0.002), weighted median (OR 1.31, 95% CI 1.08–1.59, *P* = 0.006) and outlier-corrected MR-PRESSO method (OR 1.25, 95% CI 1.07–1.46, *P* = 0.008) showed a significant association between IR and heart failure using the 53-SNPs instrument. However, this significant association did not persist after exclusion of rs1011685 (52-SNPs, Supplemental Fig. S2) or exclusion of SNPs that were correlated with HDL-C or TGs at genome-wide significance level (28-SNPs, Supplemental Fig. S3, Table S9).

For PAD, significant or suggestive evidence of relationship between IR and increased risk of PAD across sensitivity analyses was observed using 53-SNPs, 44-SNPs, or 52-SNPs instruments. The association persisted in the maximum likelihood and MR-PRESSO analyses but not in the weighted median and MR-Egger analyses when using 28-SNPs instrument; however, the precision was relatively low in MR-Egger method and no obvious pleiotropy was identified. The results for atrial fibrillation, VTE, and aortic aneurysm were consistent and robust across all sensitivity analyses whenever using 53-SNPs (Fig. 2), 52-SNPs (Supplemental Fig. S2), 44-SNPs (Supplemental Table S8), or 28-SNPs (Supplemental Fig. S3) instrument, showing no causal associations between IR and the risk of atrial fibrillation (*P* = 0.836), VTE (*P* = 0.368) and aortic aneurysm (*P* = 0.088).

### Multivariable MR analysis

**Figure 3:**
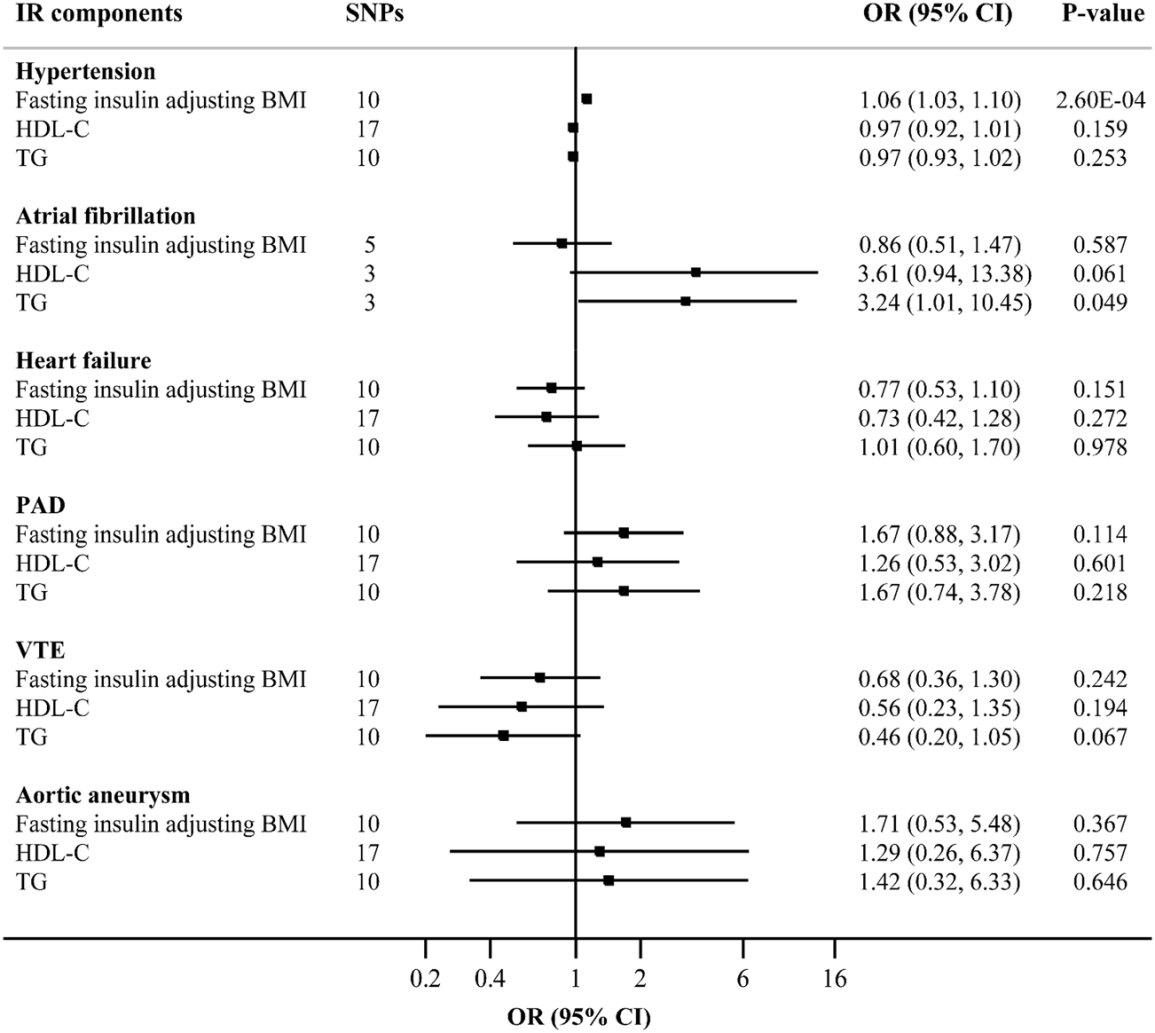
Associations of fasting insulin adjusting BMI, HDL-C and triglycerides with 6 CVD outcomes in multivariable inverse‐variance weighted model (STATA, version 13.1, URL: https://www.stata.com). *BMI* body mass index, *CI* confidence interval, *CVD* cardiovascular disease, *HDL-C* high-density lipoprotein cholesterol, *IR* insulin resistance, *OR* odds ratio, *PAD* peripheral artery disease, *VTE* venous thromboembolism.

After adjustment for the genetic correlation between different components of IR, we revealed that fasting insulin adjusted for BMI was associated with hypertension risk (OR 1.06, 95% CI 1.03–1.10, *P* = 2.60 × 10^–4^), and TG was associated with atrial fibrillation (OR 3.24, 95% CI 1.01–10.45, *P* = 0.049). No association between individual IR components and other CVDs was identified (Fig. 3).

To investigate whether the observed associations between IR and CVDs were mediated by lipid phenotypes, we further performed a multivariable MR between IR, HDL-C, and TG. After adjustment for HDL-C and TG, the causal association between IR and hypertension was partially attenuated (OR 1.02, 95% CI 1.00–1.04, *P* = 0.022), whereas the associations for heart failure and PAD were completely attenuated (Supplemental Table S10).

Multivariable MR analysis was also conducted to assess whether the associations between genetically predicted IR and CVD outcomes were mediated by BMI. The associations of IR with risk of hypertension (OR 1.06, 95% CI 1.04–1.08, *P* = 1.24 × 10^–13^) and heart failure (OR 1.22, 95% CI 1.04–1.43, *P* = 0.014) were essentially unchanged after adjustment for BMI. However, the association between IR and PAD did not persist after adjustment (Supplemental Table S11).

## Discussion

### Principal findings

This MR study demonstrated that genetically predicted IR was significantly associated with increased risk of hypertension and PAD. Furthermore, we found a suggestive association of genetically predicted IR with increased risk of heart failure. There was no evidence of causal associations of IR with atrial fibrillation, VTE and aortic aneurysm. Multivariable MR revealed that fasting insulin adjusted for BMI was associated with hypertension after adjustment for other components of IR. This suggests that fasting insulin (BMI adjusted) is the critical entity that underlies the positive association of IR and hypertension. However, no significant association between individual IR components and heart failure and PAD was identified after adjustment for other components of IR.

### Comparison with other studies

IR may increase the risk of hypertension through activating sympathetic nerve and the renin–angiotensin–aldosterone system^36^. Previous epidemiological evidence on association of IR with hypertension primarily originates from prospective cohort studies. A meta-analysis of 10 prospective observational studies showed that fasting insulin was associated with increased risk of hypertension^3^, and a more recent meta-analysis of 11 prospective studies comprised of 10,230 cases showed that homeostasis model assessment insulin resistance (HOMA-IR) was associated with 43% higher risk of incident hypertension^37^. Substantial difference on the risk of hypertension was observed between previous findings and our results (43% vs. 6%). This disparity might be attributed to systematic biases in these observational investigations, as the relationship between IR and hypertension could be frequently confounded by BMI and lipid profiles^38,39^. Associations attenuated after adjustment for BMI in meta-analysis also suggests the potential bias^37^. Our MR study provides the first evidence that the association between IR and the increased risk of hypertension is likely causal, as multiple sensitivity analyses to test the MR assumptions yielded consistent results and suggested no indication of a violation of the MR principles. The association persisted after adjustment for BMI in multivariable MR analysis, suggesting the impact of IR on hypertension risk was unlikely mediated by BMI. However, HDL-C and TG partially mediated this association. To the best of our knowledge, this is the first MR study to evaluate and corroborate IR as a causal risk factor for hypertension. Maintaining IR at normal level will contribute to the prevention of hypertension and its related disease burden in general population.

Observational studies provided inconsistent results on the association between IR and atrial fibrillation. Three prospective cohort studies^8,40,41^, using either fasting or post-glucose IR measures, reported no significant association between IR phenotype and the risk of incident atrial fibrillation in Western populations. In contrast, a more recent prospective study conducted in nondiabetic Asian populations with a median follow-up of 12.3 years showed that, HOMA-IR was significantly associated with a 1.6-fold higher risk of new-onset atrial fibrillation^4^. This discrepancy may be partly attributed to unrecognized confounding factors in prospective cohort studies, as well as population differences. Our MR analysis which can largely circumvent confounding bias showed no association between IR and atrial fibrillation, was in line with previous observational studies performed on Western populations. Since atrial fibrillation susceptibility was modulated by race and ethnicity, the role of IR for atrial fibrillation in non-European populations may require further investigation.

The association between IR and risk of heart failure was inconsistent in previous observational studies^5,9–11^. Bahrami et al. performed a large prospective cohort study (N = 6814 participants) revealed no significant association of HOMA-IR with incident heart failure during a median follow-up of 4 years^10^. Another study on glycemic parameters and incident heart failure in participants ≥ 70 years old reported significant association for fasting glucose, but not with HOMA-IR^11^. In contrast, Banerjee et al. conducted an analysis on Cardiovascular Health Study (CHS) using multiple measures of IR (fasting insulin, HOMA-IR levels, and oral glucose tolerance testing) found that IR was positively associated with the risk of incident heart failure during a median follow-up of 12 years^5^. It is noteworthy that this association was partly attenuated after adjustment for CVD risk factors such as smoking, alcohol intake, HDL-C, total cholesterol, systolic blood pressure, and waist circumference (hazard ratio [HR] 1.08; 95% CI 1.03–1.14). Hence, the discrepancy might derive from potential confounding factors and limited follow-up periods. Our MR analysis reflecting long-term exposure of IR, found that IR was a causal factor for heart failure, which was in line with the outcomes from CHS study^5^. However, performing multivariable MR to adjust for HDL-C and TGs showed attenuation of the IR effect estimates for the heart failure outcome as compared with the main univariable MR (Fig. 2), suggesting that part of the effect of IR on the heart failure outcome is mediated through these serum lipids.

Current evidence on IR and the risk of PAD is limited. It has been suggested that IR is involved in the synthesis and release of nitric oxide in endothelium^42^, and reduced endothelial nitric oxide production may factor into atherosclerosis^43^. IR status can also increase the risk of PAD through impairing fibrinolysis and promoting thrombosis and platelet aggregation^44^. A prospective analysis of CHS cohort found that IR was an independent risk factor for incident PAD during a median follow-up period of 14.1 years^6^. However, observational studies suffered from confounding and therefore conclusions were less convincing. IR had been implicated in arterial atherosclerosis process, but this association can be largely confounded by clustered expression of components of metabolic syndrome such as obesity and dyslipidemia^45^. By exploiting genetic instruments associated with IR, our findings provide further evidence of an association between IR and increased risk of PAD. However, conducting multivariable MR to adjust for genetic associations with HDL-C and TG attenuated the estimates for the PAD outcome, supporting that the effect of IR on PAD is partially mediated through serum lipids. Multivariable MR also revealed that BMI partially mediated this association (Supplemental Table S11). Therefore, strategies adopted to modify serum lipids and BMI in individuals with IR can be beneficial for lowering the burden of PAD.

Observational studies on the relationship between IR and VTE are scarce. Schouwenburg et al.^12^ performed a prospective community-based cohort study of 7,393 participants to assess HOMA-IR and fasting insulin measured IR with VTE risk. The authors demonstrated a significant association between IR and increased risk of VTE (hazard ratio [HR] 1.38; 95% CI 1.09–1.75; P = 0.007); however, this association no longer persist after adjustment for BMI (HR 1.11; 95% CI 0.85–1.43; P = 0.45)^12^. Therefore, the association for IR and VTE in this observational study is likely influenced by confounding factors. Our MR estimates which were less likely affected by confounding factors, did not show any evidence for a causal association between IR and the risk of VTE. Currently, there are few studies on IR in relation to aortic aneurysm, and our MR analysis based on relatively small sample size suggest no association. However, due to the limited number of cases, we cannot entirely rule out that our study may have overlooked a weak relationship.

### Strengths and limitations

Our study has several strengths. First, the 2-sample MR design largely avoided the limitations of confounding factors and reverse causation bias in observational studies by applying comprehensive genetic instruments for IR. Second, there was no or very limited overlap in participants from exposure GWAS and outcome datasets to minimize the type 1 error rate. Third, multiple sensitivity analyses utilized in this MR study yielded robust and consistent evidence. Fourth, our approach diminished population stratification bias by confining the study population to participants of European-descent.

However, our study is subject to several limitations. First, the probability that the IR-related genetic variants influence the risk of CVD through potential unknown pathways than through IR exposure cannot be entirely ruled out. However, the results were consistent across all sensitivity analyses and persisted after excluding SNPs that were significantly associated with BMI or lipids. Second, since there is no large-scale GWAS on gold-standard measures of IR, we used three traits of IR (higher fasting insulin levels adjusted for BMI, lower HDL-C and higher TGs levels) to mark IR phenotype according to a GWAS meta-analysis. The proxy IR based on 3 traits may neglect other traits that were associated with IR and weaken the clinical relevance. Nonetheless, the strong association between identified SNPs and IR had been validated by the gold standard measures of insulin sensitivity in several independent cohorts^17^. Third, as the proportion of variation in IR explained by the genetic variants was not available in exposure GWAS, we cannot calculate the statistical power of our MR analyses. However, previous MR analysis utilizing the same genetic instruments has shown significant associations of IR with other CVDs^15^. Moreover, the outcome GWASs with large sample size which were included in our MR study could provide high statistical power. Fourth, our MR study was performed using data from European-ancestry population-based studies and may not generalize to non-Europeans. Therefore, further investigations on the association between IR and the risk of CVD in non-Europeans are warranted.

## Conclusions

This MR study provided evidence to show that genetically determined IR increases the risk of hypertension and PAD. Current evidence does not support causal associations between IR and atrial fibrillation, VTE and aortic aneurysm. Future studies with large sample sizes are warranted to determine causal association between IR and risk of aortic aneurysm.

### Supplementary Information

Supplementary Information 1.

Supplementary Figures.

Supplementary Tables.

## Data Availability

All data used in this study are included in the article or uploaded as supplementary materials. There are no additional, unpublished data available from this study. Software codes and data are available upon request.
