# Supplementary material for: Mendelian randomization study on insulin resistance and risk of hypertension and cardiovascular disease
Source: Sci Rep. 2024 Mar 14;14:6191. doi: 10.1038/s41598-023-46983-3 (PMC10940700; doi:10.1038/s41598-023-46983-3)
Supplement: Supplementary file 2 — Supplementary Figures. [file 41598_2023_46983_MOESM2_ESM.docx]

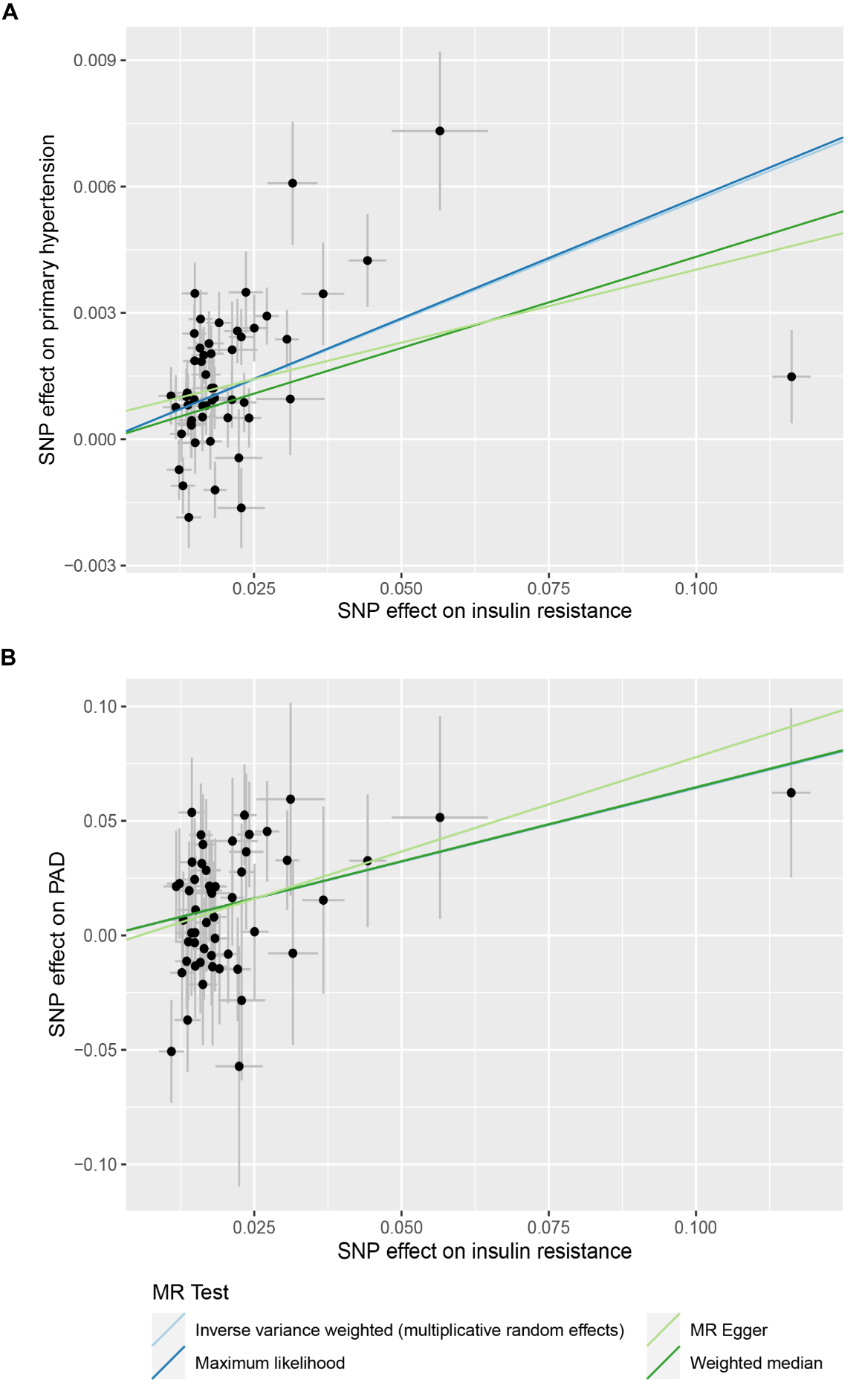
­­

**Supplemental Figure S1.** Associations between SNPs correlated to IR and risk of (**A**) hypertension and (**B**) PAD. Per allele associations with exposure plotted against per allele associations with outcome (vertical and horizontal black lines around points indicate 95% confidence interval for each polymorphism), generated by RStudio (version 3.6.1, URL:
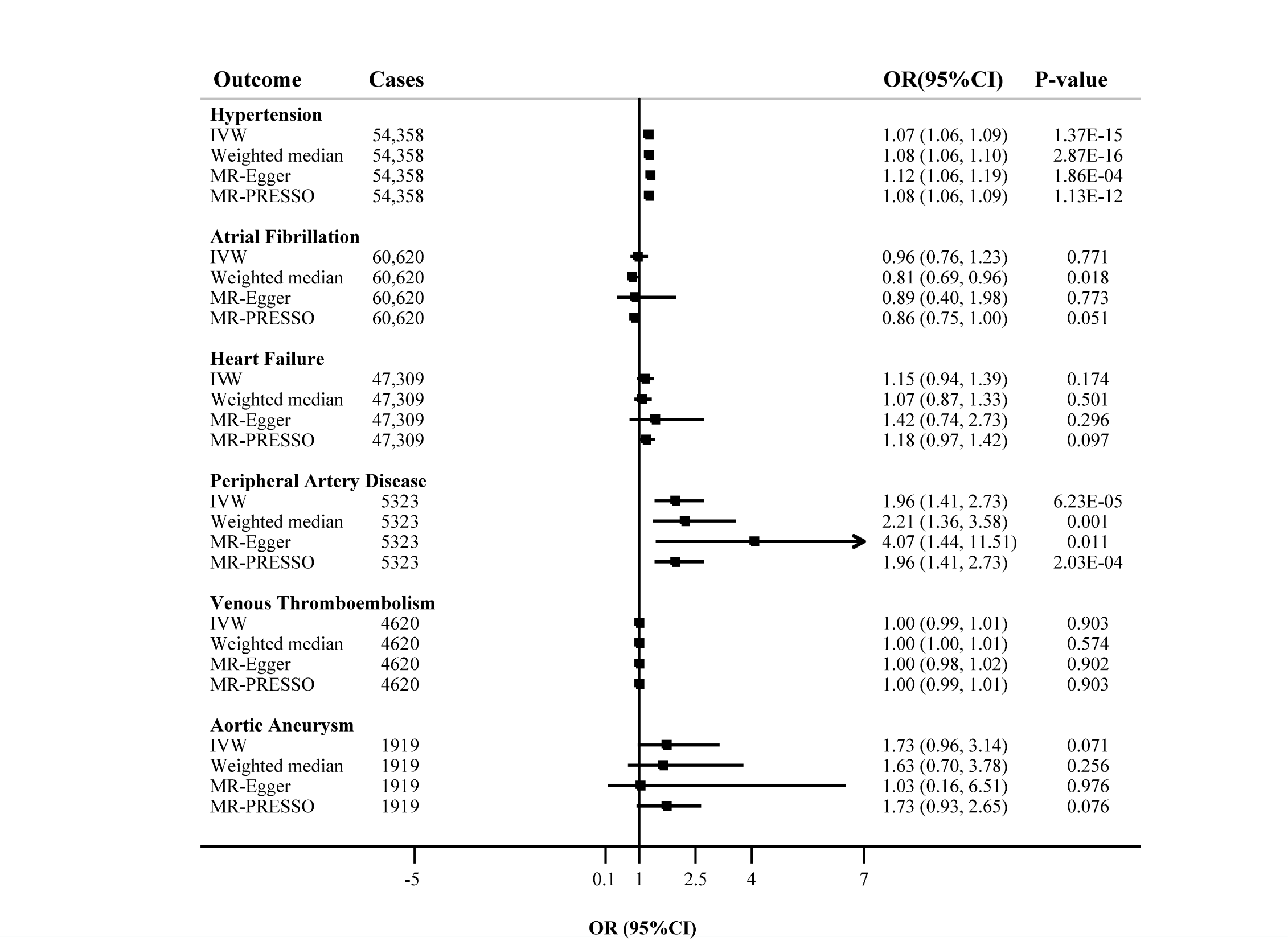
https://www.r-project.org/).

**Supplemental Figure S2.** Causal associations of genetically predicted IR with CVD outcomes based on 52-SNPs instrument after excluding rs1011685. ORs are per 1 SD change in IR exposure (STATA, version 13.1, URL: https://www.stata.com). CI, confidence interval; CVD, cardiovascular disease; IVW, inverse-variance weighted; IR, insulin resistance; MR-PRESSO, MR pleiotropy residual sum and outlier test; OR, odds ratio.


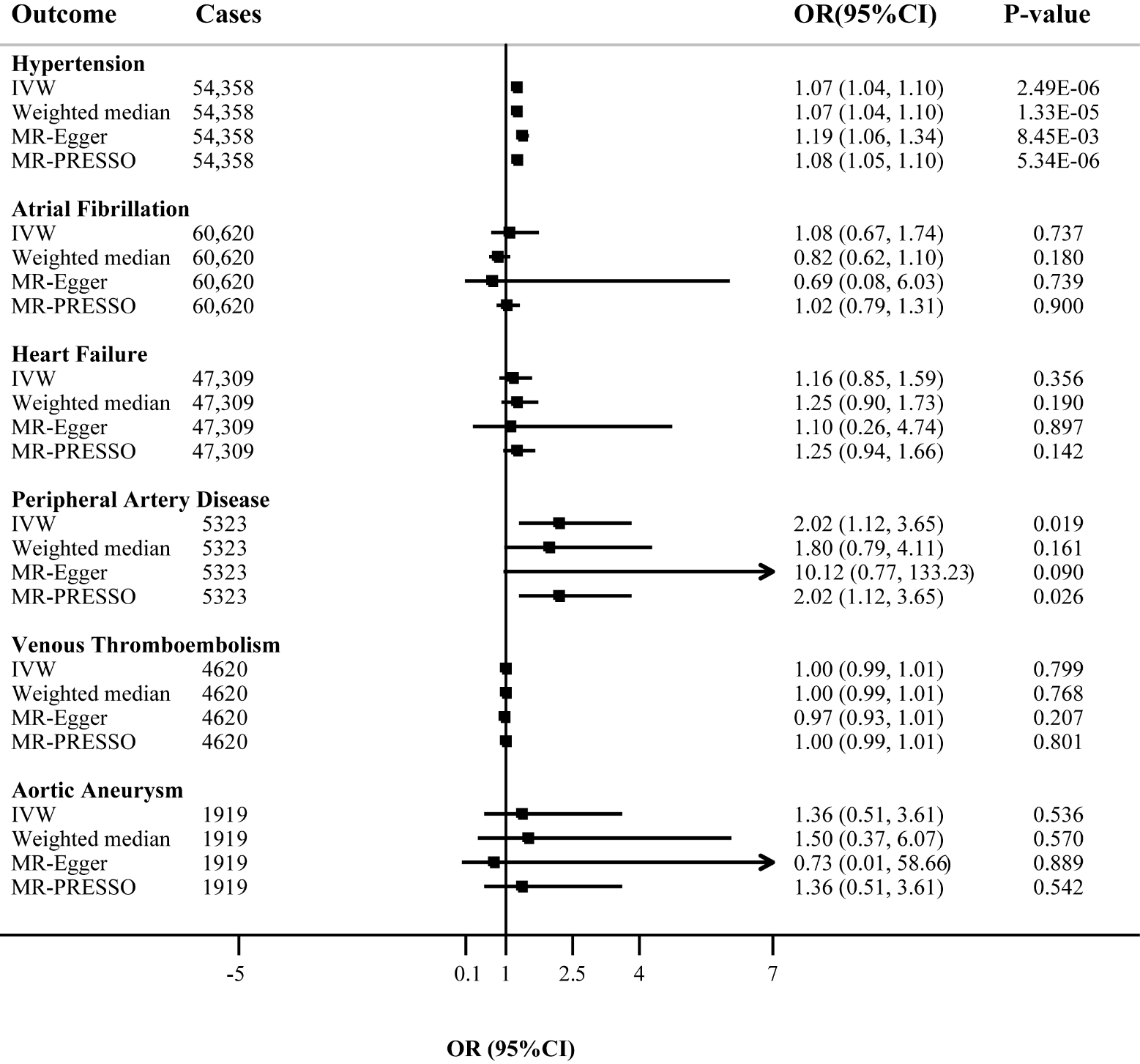


**Supplemental Figure S3.** Causal associations of genetically predicted IR with CVD outcomes based on 28-SNPs instrument after excluding SNPs that were related to lipids (STATA, version 13.1, URL: https://www.stata.com). ORs are per 1 SD change in IR exposure. CI, confidence interval; CVD, cardiovascular disease; IVW, inverse-variance weighted; IR, insulin resistance; MR-PRESSO, MR pleiotropy residual sum and outlier test; OR, odds ratio.
