# Supplementary material for: Mendelian randomization study on insulin resistance and risk of hypertension and cardiovascular disease
Source: Sci Rep. 2024 Mar 14;14:6191. doi: 10.1038/s41598-023-46983-3 (PMC10940700; doi:10.1038/s41598-023-46983-3)
Supplement: Supplementary file 3 — Supplementary Tables. [file 41598_2023_46983_MOESM3_ESM.docx]

**Insulin Resistance and Risk of Hypertension, Atrial Fibrillation, and Other Cardiovascular Disease: A Mendelian Randomization Study**

Zhimin Yu, Fangfang Zhang, Yanggan Wang

Online Supplement

**Supplemental Table S1.** Definitions of CVD outcomes in this MR study.

**Supplemental Table S2**. Description of the studies and datasets included in the Mendelian randomization analyses.

**Supplemental Table S3:** All 53 SNPs associated with insulin resistance phenotypic pattern identified by Lotta et al used as genetic instruments.

**Supplemental Table S4:** 52 SNPs associated with insulin resistance phenotypic pattern after excluding rs1011685.

**Supplemental Table S5:** 28 SNPs associated with insulin resistance phenotypic pattern but not correlated to TGs or HDL-C at genome-wide significance.

**Supplemental Table S6:** 44 SNPs associated with insulin resistance phenotypic pattern but not correlated to BMI at significance level (*p* < 0.001).

**Supplementary Table S7:** Effect estimates for the associations of the selected variants with insulin resistance and 6 cardiovascular disease outcomes.

**Supplemental Table S8:** Sensitivity analyses of the Mendelian randomization study on insulin resistance and cardiovascular disease.

**Supplemental Table S9:** Inverse-variance weighted MR estimates between insulin resistance and cardiovascular diseases, after excluding SNPs that are nominally associated with BMI.

**Supplemental Table S10:** Multivariable Mendelian randomization associations of insulin resistance with CVD risk adjusting for lipid traits.

**Supplemental Table S11:** Multivariable Mendelian randomization associations of insulin resistance with CVD risk adjusting for BMI.

**Supporting Information**

**Supplemental Table S1.** Definitions of CVD outcomes in this MR study.

| **Cohort** | **Definition of Cardiovascular Disease** |
| --- | --- |
| **Hypertension^1^** | ICD-10: I10 |
| **Atrial Fibrillation^2^** |  |
| HUNT | Combination of hospital, out-patient, and emergency room discharge diagnoses (ICD-9 and ICD-10) |
| deCODE | ICD-10: I48 and ICD-9:427.3 |
| MGI | ICD-9: 427.31 |
| DiscovEHR | ICD-10: I48 |
| UK Biobank | ICD-9:427.3 and ICD-10:I48 |
| AFGen Consortium | Adjudication of atrial fibrillation included either documented atrial fibrillation on an electrocardiogram and/or one in-patient or two out-patient diagnoses of atrial fibrillation (data obtained from the Authors) |
| **Heart failure^3^** | Combination of self-reported heart failure, medical records, ICD-9: 428 and subcodes, ICD-10: I50 and subcodes |
| **Peripheral Artery Disease^4^** | ICD-8: 25006\|4402\|4439, ICD-9: 4402\|4439 and ICD-10: E105\|E115\|E125\|E135\|E145\|I702\|I739 |
| **Venous Thromboembolism^1^** | UK Biobank codes: 1068, 1093, 1094; ICD-9: 415.1, 416.2, 451-453; ICD-10: I26, I80-I82, I26,I80-I82 |
| **Aortic aneurysm^4^** | ICD-8: 0930\|441, ICD-9: 441\|0930A and ICD-10: I71 |
| ICD: international classification of diseases, 8^th^, 9^th^ and 10^th^ Revision  From: 1. Neale-Lab. UK Biobank GWAS 2018. Available from: http://www.nealelab.is/uk-biobank/.  2. Nielsen JB, Thorolfsdottir RB, Fritsche LG, et al. Biobank-driven genomic discovery yields new insight into atrial fibrillation biology. Nat Genet. 2018;50:1234-1239.  3. Shah S, Henry A, Roselli C, et al. Genome-wide association and Mendelian randomisation analysis provide insights into the pathogenesis of heart failure. Nat Commun. 2020;11(1):163.  4. FinnGen (2020). FinnGen Documentation of R4 release. https://finngen.gitbook.io/documentation/. Accessed March 10, 2021. | |

**Supplemental Table S2.** Description of the studies and datasets included in the Mendelian randomization analyses.

| Phenotype | Consortium | No. of cases | No. of controls | Ancestry | Use in this MR study | Adjustments |
| --- | --- | --- | --- | --- | --- | --- |
| Insulin resistance |  |  |  |  |  |  |
| Fasting insulin | MAGIC [22] | NA | 188,577 | European | Exposure | Age, sex, BMI, cohort |
| HDL-C and  triglycerides | GLGC [23] | NA | 188,577 | European | Exposure | Age, sex, PCs |
| Outcomes |  |  |  |  |  |  |
| Hypertension^a^ | UK Biobank (Neale Lab) [18] | 54,358 | 408,652 | European | Outcome | Sex |
| Atrial fibrillation | Meta-analyzed GWAS^b^ [19] | 60,620 | 970,216 | European | Outcome | Age, sex, PCs |
| Heart failure | HERMES Consortium [20] | 47,309 | 930,014 | European | Outcome | Age, sex, genotyping array, 10 PCs |
| PAD | FinnGen (release 4) [21] | 5323 | 167,843 | European | Outcome | Age, sex, 10 PCs, genotyping batch |
| VTE^a^ | UK Biobank (Neale Lab) [18] | 4620 | 356,574 | European | Outcome | Sex |
| Aortic aneurysm | FinnGen (release 4) [21] | 1919 | 167843 | European | Outcome | Age, sex, 10 PCs, genotyping batch |

BMI, body mass index; GLGC, Global Lipids Genetics Consortium; GWAS, genome-wide association study; HDL-C, high-density lipoprotein cholesterol; HERMES, Heart Failure Molecular Epidemiology for Therapeutic Targets Consortium; MAGIC, Meta-Analyses of Glucose and Insulin-related traits Consortium; MR, Mendelian randomization; PAD, peripheral artery disease; PCs: principal components; VTE, venous thromboembolism; ^a^Summary-level data for these outcomes were accessed through the MR-Base platform; ^b^The GWAS consortium comprised of six studies (The Nord-Trøndelag Health Study (HUNT), deCODE, the Michigan Genomics Initiative (MGI), DiscovEHR, UK Biobank, and the AFGen Consortium).

**Supplemental Table S3:** All 53 SNPs associated with insulin resistance phenotypic pattern identified by Lotta et al used as genetic instruments.

| **SNP** | **Nearest gene** |  | **Position** | **EA** | **OA** | **EAF** |
| --- | --- | --- | --- | --- | --- | --- |
| rs17386142 | *DMRTA2* |  | chr1:50815783 | C | T | 0.9274 |
| rs11577194 | *CSF1* |  | chr1:110500175 | T | C | 0.4789 |
| rs9425291 | *DNM3* |  | chr1:172312769 | A | G | 0.4288 |
| rs4846565 | *RNU5F-1/LYPLAL1* |  | chr1:219722104 | G | A | 0.6939 |
| rs2249105 | *CEP68* |  | chr2:65287896 | A | G | 0.6227 |
| rs492400 | *USP37* |  | chr2:219349752 | T | C | 0.6029 |
| rs308971 | *SYN2/PPARG* |  | chr3:12116620 | G | A | 0.1385 |
| rs3864041 | *COL6A4P1* |  | chr3:15185634 | T | C | 0.6227 |
| rs9881942 | *ADCY5* |  | chr3:123082416 | A | G | 0.4354 |
| rs6822892 | *PDGFC* |  | chr4:157734675 | A | G | 0.6464 |
| rs4976033 | *PIK3R1* |  | chr5:67714246 | G | A | 0.3799 |
| rs6887914 | *MCC* |  | chr5:112711486 | C | T | 0.781 |
| rs1045241 | *TNFAIP8* |  | chr5:118729286 | C | T | 0.7427 |
| rs2434612 | *EBF1* |  | chr5:158022041 | G | A | 0.2071 |
| rs966544 | *CPEB4* |  | chr5:173350405 | G | A | 0.3074 |
| rs12525532 | *ANKS1A* |  | chr6:35004819 | T | C | 0.3958 |
| rs9492443 | *L3MBTL3* |  | chr6:130398731 | C | T | 0.7639 |
| rs17169104 | *MEOX2* |  | chr7:15883727 | G | C | 0.3417 |
| rs4738141 | *EYA1* |  | chr8:72469742 | G | A | 0.2493 |
| rs498313 | *MIR548H3* |  | chr9:78034169 | A | G | 0.6913 |
| rs11231693 | *MACROD1* |  | chr11:63862612 | A | G | 0.0606 |
| rs17402950 | *ATF7IP* |  | chr12:14571671 | G | A | 0.0554 |
| rs718314 | *ITPR2* |  | chr12:26453283 | G | A | 0.2348 |
| rs7323406 | *ANKRD10* |  | chr13:111628195 | A | G | 0.2784 |
| rs7176058 | *C15orf54* |  | chr15:39464167 | A | G | 0.8364 |
| rs8032586 | *LOC100287559* |  | chr15:73081067 | C | T | 0.8813 |
| rs754814 | *ZMYND15* |  | chr17:4657034 | T | C | 0.7309 |
| rs6066149 | *EYA2* |  | chr20:45602638 | G | A | 0.7612 |
| rs683135 | *MACF1* |  | chr1:39895460 | A | G | 0.2678 |
| rs10195252 | *COBLL1/GRB14* |  | chr2:165513091 | T | C | 0.5818 |
| rs2943645 | *IRS1* |  | chr2:227099180 | T | C | 0.6227 |
| rs295449 | *KLHL18* |  | chr3:47375955 | A | G | 0.595 |
| rs11130329 | *TMEM110-MUSTN1* |  | chr3:52896855 | A | C | 0.8641 |
| rs645040 | *MSL2* |  | chr3:135926622 | T | G | 0.7691 |
| rs2699429 | *DOK7* |  | chr4:3480136 | C | T | 0.4063 |
| rs3822072 | *FAM13A* |  | chr4:89741269 | A | G | 0.4881 |
| rs4865796 | *ARL15/FST* |  | chr5:53272664 | A | G | 0.7071 |
| rs459193 | *ANKRD55* |  | chr5:55806751 | G | A | 0.715 |
| rs6937438 | *LOC100132354* |  | chr6:43815364 | A | G | 0.7084 |
| rs2745353 | *RSPO3* |  | chr6:127452935 | T | C | 0.4789 |
| rs3861397 | *LOC645434* |  | chr6:139828916 | G | A | 0.3417 |
| rs972283 | *KLF14* |  | chr7:130466854 | G | A | 0.5435 |
| rs2126259 | *PPP1R3B* |  | chr8:9185146 | T | C | 0.0910 |
| rs1011685 | *LPL* |  | chr8:19830769 | C | T | 0.8773 |
| rs7005992 | *TRIB1* |  | chr8:126528955 | C | G | 0.1359 |
| rs10995441 | *NRBF2* |  | chr10:64869239 | G | T | 0.2361 |
| rs7973683 | *CCDC92/DNAH10* |  | chr12:124449223 | C | A | 0.6385 |
| rs7227237 | *LIPG* |  | chr18:47174679 | C | T | 0.7678 |
| rs8101064 | *INSR* |  | chr19:7293119 | T | C | 0.0435 |
| rs4804833 | *MAP2K7* |  | chr19:7970635 | A | G | 0.4103 |
| rs4804311 | *MYO1F* |  | chr19:8615589 | A | G | 0.8905 |
| rs731839 | *PEPD* |  | chr19:33899065 | G | A | 0.3417 |
| rs132985 | *PLA2G6* |  | chr22:38563471 | C | T | 0.5646 |

**Supplemental Table S4:** 52 SNPs associated with insulin resistance phenotypic pattern after excluding rs1011685.

| **SNP** | **Nearest gene** |  | **Position** | **EA** | **OA** | **EAF** |
| --- | --- | --- | --- | --- | --- | --- |
| rs17386142 | *DMRTA2* |  | chr1:50815783 | C | T | 0.9274 |
| rs11577194 | *CSF1* |  | chr1:110500175 | T | C | 0.4789 |
| rs9425291 | *DNM3* |  | chr1:172312769 | A | G | 0.4288 |
| rs4846565 | *RNU5F-1/LYPLAL1* |  | chr1:219722104 | G | A | 0.6939 |
| rs2249105 | *CEP68* |  | chr2:65287896 | A | G | 0.6227 |
| rs492400 | *USP37* |  | chr2:219349752 | T | C | 0.6029 |
| rs308971 | *SYN2/PPARG* |  | chr3:12116620 | G | A | 0.1385 |
| rs3864041 | *COL6A4P1* |  | chr3:15185634 | T | C | 0.6227 |
| rs9881942 | *ADCY5* |  | chr3:123082416 | A | G | 0.4354 |
| rs6822892 | *PDGFC* |  | chr4:157734675 | A | G | 0.6464 |
| rs4976033 | *PIK3R1* |  | chr5:67714246 | G | A | 0.3799 |
| rs6887914 | *MCC* |  | chr5:112711486 | C | T | 0.781 |
| rs1045241 | *TNFAIP8* |  | chr5:118729286 | C | T | 0.7427 |
| rs2434612 | *EBF1* |  | chr5:158022041 | G | A | 0.2071 |
| rs966544 | *CPEB4* |  | chr5:173350405 | G | A | 0.3074 |
| rs12525532 | *ANKS1A* |  | chr6:35004819 | T | C | 0.3958 |
| rs9492443 | *L3MBTL3* |  | chr6:130398731 | C | T | 0.7639 |
| rs17169104 | *MEOX2* |  | chr7:15883727 | G | C | 0.3417 |
| rs4738141 | *EYA1* |  | chr8:72469742 | G | A | 0.2493 |
| rs498313 | *MIR548H3* |  | chr9:78034169 | A | G | 0.6913 |
| rs11231693 | *MACROD1* |  | chr11:63862612 | A | G | 0.0606 |
| rs17402950 | *ATF7IP* |  | chr12:14571671 | G | A | 0.0554 |
| rs718314 | *ITPR2* |  | chr12:26453283 | G | A | 0.2348 |
| rs7323406 | *ANKRD10* |  | chr13:111628195 | A | G | 0.2784 |
| rs7176058 | *C15orf54* |  | chr15:39464167 | A | G | 0.8364 |
| rs8032586 | *LOC100287559* |  | chr15:73081067 | C | T | 0.8813 |
| rs754814 | *ZMYND15* |  | chr17:4657034 | T | C | 0.7309 |
| rs6066149 | *EYA2* |  | chr20:45602638 | G | A | 0.7612 |
| rs683135 | *MACF1* |  | chr1:39895460 | A | G | 0.2678 |
| rs10195252 | *COBLL1/GRB14* |  | chr2:165513091 | T | C | 0.5818 |
| rs2943645 | *IRS1* |  | chr2:227099180 | T | C | 0.6227 |
| rs295449 | *KLHL18* |  | chr3:47375955 | A | G | 0.595 |
| rs11130329 | *TMEM110-MUSTN1* |  | chr3:52896855 | A | C | 0.8641 |
| rs645040 | *MSL2* |  | chr3:135926622 | T | G | 0.7691 |
| rs2699429 | *DOK7* |  | chr4:3480136 | C | T | 0.4063 |
| rs3822072 | *FAM13A* |  | chr4:89741269 | A | G | 0.4881 |
| rs4865796 | *ARL15/FST* |  | chr5:53272664 | A | G | 0.7071 |
| rs459193 | *ANKRD55* |  | chr5:55806751 | G | A | 0.715 |
| rs6937438 | *LOC100132354* |  | chr6:43815364 | A | G | 0.7084 |
| rs2745353 | *RSPO3* |  | chr6:127452935 | T | C | 0.4789 |
| rs3861397 | *LOC645434* |  | chr6:139828916 | G | A | 0.3417 |
| rs972283 | *KLF14* |  | chr7:130466854 | G | A | 0.5435 |
| rs2126259 | *PPP1R3B* |  | chr8:9185146 | T | C | 0.0910 |
| rs7005992 | *TRIB1* |  | chr8:126528955 | C | G | 0.1359 |
| rs10995441 | *NRBF2* |  | chr10:64869239 | G | T | 0.2361 |
| rs7973683 | *CCDC92/DNAH10* |  | chr12:124449223 | C | A | 0.6385 |
| rs7227237 | *LIPG* |  | chr18:47174679 | C | T | 0.7678 |
| rs8101064 | *INSR* |  | chr19:7293119 | T | C | 0.0435 |
| rs4804833 | *MAP2K7* |  | chr19:7970635 | A | G | 0.4103 |
| rs4804311 | *MYO1F* |  | chr19:8615589 | A | G | 0.8905 |
| rs731839 | *PEPD* |  | chr19:33899065 | G | A | 0.3417 |
| rs132985 | *PLA2G6* |  | chr22:38563471 | C | T | 0.5646 |

**Supplemental Table S5:** 28 SNPs associated with insulin resistance phenotypic pattern but not correlated to TGs or HDL-C at genome-wide significance.

| **SNP** | **Nearest gene** |  | **Position** | **EA** | **OA** | **EAF** |
| --- | --- | --- | --- | --- | --- | --- |
| rs17386142 | *DMRTA2* |  | chr1:50815783 | C | T | 0.9274 |
| rs11577194 | *CSF1* |  | chr1:110500175 | T | C | 0.4789 |
| rs9425291 | *DNM3* |  | chr1:172312769 | A | G | 0.4288 |
| rs4846565 | *RNU5F-1/LYPLAL1* |  | chr1:219722104 | G | A | 0.6939 |
| rs2249105 | *CEP68* |  | chr2:65287896 | A | G | 0.6227 |
| rs492400 | *USP37* |  | chr2:219349752 | T | C | 0.6029 |
| rs308971 | *SYN2/PPARG* |  | chr3:12116620 | G | A | 0.1385 |
| rs3864041 | *COL6A4P1* |  | chr3:15185634 | T | C | 0.6227 |
| rs9881942 | *ADCY5* |  | chr3:123082416 | A | G | 0.4354 |
| rs6822892 | *PDGFC* |  | chr4:157734675 | A | G | 0.6464 |
| rs4976033 | *PIK3R1* |  | chr5:67714246 | G | A | 0.3799 |
| rs6887914 | *MCC* |  | chr5:112711486 | C | T | 0.781 |
| rs1045241 | *TNFAIP8* |  | chr5:118729286 | C | T | 0.7427 |
| rs2434612 | *EBF1* |  | chr5:158022041 | G | A | 0.2071 |
| rs966544 | *CPEB4* |  | chr5:173350405 | G | A | 0.3074 |
| rs12525532 | *ANKS1A* |  | chr6:35004819 | T | C | 0.3958 |
| rs9492443 | *L3MBTL3* |  | chr6:130398731 | C | T | 0.7639 |
| rs17169104 | *MEOX2* |  | chr7:15883727 | G | C | 0.3417 |
| rs4738141 | *EYA1* |  | chr8:72469742 | G | A | 0.2493 |
| rs498313 | *MIR548H3* |  | chr9:78034169 | A | G | 0.6913 |
| rs11231693 | *MACROD1* |  | chr11:63862612 | A | G | 0.0606 |
| rs17402950 | *ATF7IP* |  | chr12:14571671 | G | A | 0.0554 |
| rs718314 | *ITPR2* |  | chr12:26453283 | G | A | 0.2348 |
| rs7323406 | *ANKRD10* |  | chr13:111628195 | A | G | 0.2784 |
| rs7176058 | *C15orf54* |  | chr15:39464167 | A | G | 0.8364 |
| rs8032586 | *LOC100287559* |  | chr15:73081067 | C | T | 0.8813 |
| rs754814 | *ZMYND15* |  | chr17:4657034 | T | C | 0.7309 |
| rs6066149 | *EYA2* |  | chr20:45602638 | G | A | 0.7612 |

**Supplemental Table S6:** 44 SNPs associated with insulin resistance phenotypic pattern but not correlated to BMI at significance level (*p* < 0.001).

| **SNP** | **Nearest gene** |  | **Position** | **EA** | **OA** | **EAF** |
| --- | --- | --- | --- | --- | --- | --- |
| rs17386142 | *DMRTA2* |  | chr1:50815783 | C | T | 0.9274 |
| rs11577194 | *CSF1* |  | chr1:110500175 | T | C | 0.4789 |
| rs9425291 | *DNM3* |  | chr1:172312769 | A | G | 0.4288 |
| rs4846565 | *RNU5F-1/LYPLAL1* |  | chr1:219722104 | G | A | 0.6939 |
| rs2249105 | *CEP68* |  | chr2:65287896 | A | G | 0.6227 |
| rs308971 | *SYN2/PPARG* |  | chr3:12116620 | G | A | 0.1385 |
| rs3864041 | *COL6A4P1* |  | chr3:15185634 | T | C | 0.6227 |
| rs6822892 | *PDGFC* |  | chr4:157734675 | A | G | 0.6464 |
| rs4976033 | *PIK3R1* |  | chr5:67714246 | G | A | 0.3799 |
| rs6887914 | *MCC* |  | chr5:112711486 | C | T | 0.781 |
| rs1045241 | *TNFAIP8* |  | chr5:118729286 | C | T | 0.7427 |
| rs2434612 | *EBF1* |  | chr5:158022041 | G | A | 0.2071 |
| rs12525532 | *ANKS1A* |  | chr6:35004819 | T | C | 0.3958 |
| rs9492443 | *L3MBTL3* |  | chr6:130398731 | C | T | 0.7639 |
| rs17169104 | *MEOX2* |  | chr7:15883727 | G | C | 0.3417 |
| rs4738141 | *EYA1* |  | chr8:72469742 | G | A | 0.2493 |
| rs498313 | *MIR548H3* |  | chr9:78034169 | A | G | 0.6913 |
| rs17402950 | *ATF7IP* |  | chr12:14571671 | G | A | 0.0554 |
| rs718314 | *ITPR2* |  | chr12:26453283 | G | A | 0.2348 |
| rs7323406 | *ANKRD10* |  | chr13:111628195 | A | G | 0.2784 |
| rs7176058 | *C15orf54* |  | chr15:39464167 | A | G | 0.8364 |
| rs754814 | *ZMYND15* |  | chr17:4657034 | T | C | 0.7309 |
| rs6066149 | *EYA2* |  | chr20:45602638 | G | A | 0.7612 |
| rs683135 | *MACF1* |  | chr1:39895460 | A | G | 0.2678 |
| rs295449 | *KLHL18* |  | chr3:47375955 | A | G | 0.595 |
| rs11130329 | *TMEM110-MUSTN1* |  | chr3:52896855 | A | C | 0.8641 |
| rs2699429 | *DOK7* |  | chr4:3480136 | C | T | 0.4063 |
| rs3822072 | *FAM13A* |  | chr4:89741269 | A | G | 0.4881 |
| rs459193 | *ANKRD55* |  | chr5:55806751 | G | A | 0.715 |
| rs6937438 | *LOC100132354* |  | chr6:43815364 | A | G | 0.7084 |
| rs2745353 | *RSPO3* |  | chr6:127452935 | T | C | 0.4789 |
| rs3861397 | *LOC645434* |  | chr6:139828916 | G | A | 0.3417 |
| rs972283 | *KLF14* |  | chr7:130466854 | G | A | 0.5435 |
| rs2126259 | *PPP1R3B* |  | chr8:9185146 | T | C | 0.0910 |
| rs1011685 | *LPL* |  | chr8:19830769 | C | T | 0.8773 |
| rs7005992 | *TRIB1* |  | chr8:126528955 | C | G | 0.1359 |
| rs10995441 | *NRBF2* |  | chr10:64869239 | G | T | 0.2361 |
| rs7227237 | *LIPG* |  | chr18:47174679 | C | T | 0.7678 |
| rs8101064 | *INSR* |  | chr19:7293119 | T | C | 0.0435 |
| rs4804833 | *MAP2K7* |  | chr19:7970635 | A | G | 0.4103 |
| rs4804311 | *MYO1F* |  | chr19:8615589 | A | G | 0.8905 |
| rs731839 | *PEPD* |  | chr19:33899065 | G | A | 0.3417 |
| rs132985 | *PLA2G6* |  | chr22:38563471 | C | T | 0.5646 |

**Supplementary Table S7:** Effect estimates for the associations of the selected variants with insulin resistance and 6 cardiovascular disease outcomes.

|  |  |  |  |  | **Insulin resistance results** | | | **CVD results** | | |
| --- | --- | --- | --- | --- | --- | --- | --- | --- | --- | --- |
| **SNP** | **Chr** | **Pos** | **Outcome** | **EA/OA** | **Beta** | **SE** | **P-value** | **Beta** | **SE** | **P-value** |
| rs11577194 | 1 | 110500175 | Primary hypertension | T/C | 0.0135 | 0.0000 | 2.91E-12 | 0.0010 | 0.0007 | 0.1300 |
| rs17386142 | 1 | 50815783 | Primary hypertension | C/T | 0.0224 | 0.0000 | 1.10E-08 | -0.0004 | 0.0012 | 0.7000 |
| rs4846565 | 1 | 219722104 | Primary hypertension | G/A | 0.0163 | 0.0000 | 2.74E-15 | 0.0008 | 0.0007 | 0.2600 |
| rs683135 | 1 | 39895460 | Primary hypertension | A/G | 0.0191 | 0.0000 | 5.55E-19 | 0.0028 | 0.0007 | 0.0001 |
| rs9425291 | 1 | 172312769 | Primary hypertension | A/G | 0.0149 | 0.0000 | 3.43E-14 | 0.0019 | 0.0007 | 0.0055 |
| rs10195252 | 2 | 165513091 | Primary hypertension | T/C | 0.0272 | 0.0000 | 2.54E-40 | 0.0029 | 0.0007 | 0.0000 |
| rs2249105 | 2 | 65287896 | Primary hypertension | A/G | 0.0159 | 0.0000 | 1.21E-15 | 0.0029 | 0.0007 | 0.0000 |
| rs2943645 | 2 | 227099180 | Primary hypertension | T/C | 0.0306 | 0.0000 | 2.02E-52 | 0.0024 | 0.0007 | 0.0006 |
| rs492400 | 2 | 219349752 | Primary hypertension | T/C | 0.0130 | 0.0000 | 1.01E-10 | -0.0011 | 0.0007 | 0.1000 |
| rs11130329 | 3 | 52896855 | Primary hypertension | A/C | 0.0228 | 0.0000 | 5.64E-09 | -0.0016 | 0.0010 | 0.0870 |
| rs295449 | 3 | 47375955 | Primary hypertension | A/G | 0.0144 | 0.0000 | 1.92E-10 | 0.0004 | 0.0007 | 0.5100 |
| rs308971 | 3 | 12116620 | Primary hypertension | G/A | 0.0236 | 0.0000 | 2.64E-16 | 0.0035 | 0.0010 | 0.0003 |
| rs3864041 | 3 | 15185634 | Primary hypertension | T/C | 0.0109 | 0.0000 | 1.32E-07 | 0.0010 | 0.0007 | 0.1300 |
| rs645040 | 3 | 135926622 | Primary hypertension | T/G | 0.0250 | 0.0000 | 6.33E-26 | 0.0026 | 0.0008 | 0.0009 |
| rs9881942 | 3 | 123082416 | Primary hypertension | A/G | 0.0127 | 0.0000 | 5.90E-11 | 0.0001 | 0.0007 | 0.8500 |
| rs2699429 | 4 | 3480136 | Primary hypertension | C/T | 0.0165 | 0.0000 | 1.30E-15 | 0.0020 | 0.0007 | 0.0032 |
| rs3822072 | 4 | 89741269 | Primary hypertension | A/G | 0.0213 | 0.0000 | 7.72E-27 | 0.0009 | 0.0007 | 0.1600 |
| rs6822892 | 4 | 157734675 | Primary hypertension | A/G | 0.0182 | 0.0000 | 1.52E-18 | 0.0012 | 0.0007 | 0.0850 |
| rs1045241 | 5 | 118729286 | Primary hypertension | C/T | 0.0137 | 0.0000 | 4.62E-10 | 0.0011 | 0.0008 | 0.1400 |
| rs2434612 | 5 | 158022041 | Primary hypertension | G/A | 0.0168 | 0.0000 | 6.31E-12 | 0.0015 | 0.0008 | 0.0620 |
| rs459193 | 5 | 55806751 | Primary hypertension | G/A | 0.0222 | 0.0000 | 1.53E-23 | 0.0026 | 0.0008 | 0.0008 |
| rs4865796 | 5 | 53272664 | Primary hypertension | A/G | 0.0158 | 0.0000 | 4.30E-14 | 0.0022 | 0.0007 | 0.0027 |
| rs4976033 | 5 | 67714246 | Primary hypertension | G/A | 0.0169 | 0.0000 | 2.39E-15 | 0.0008 | 0.0007 | 0.2500 |
| rs6887914 | 5 | 112711486 | Primary hypertension | C/T | 0.0144 | 0.0000 | 2.86E-09 | 0.0004 | 0.0008 | 0.6500 |
| rs966544 | 5 | 173350405 | Primary hypertension | G/A | 0.0139 | 0.0000 | 3.74E-11 | -0.0019 | 0.0007 | 0.0100 |
| rs12525532 | 6 | 35004819 | Primary hypertension | T/C | 0.0149 | 0.0000 | 1.26E-13 | 0.0025 | 0.0007 | 0.0003 |
| rs2745353 | 6 | 127452935 | Primary hypertension | T/C | 0.0184 | 0.0000 | 5.78E-21 | -0.0012 | 0.0007 | 0.0700 |
| rs3861397 | 6 | 139828916 | Primary hypertension | G/A | 0.0206 | 0.0000 | 6.27E-24 | 0.0005 | 0.0007 | 0.4700 |
| rs6937438 | 6 | 43815364 | Primary hypertension | A/G | 0.0150 | 0.0000 | 4.37E-12 | 0.0035 | 0.0007 | 0.0000 |
| rs9492443 | 6 | 130398731 | Primary hypertension | C/T | 0.0144 | 0.0000 | 5.87E-11 | 0.0003 | 0.0008 | 0.6600 |
| rs17169104 | 7 | 15883727 | Primary hypertension | G/C | 0.0183 | 0.0000 | 1.23E-13 | 0.0010 | 0.0007 | 0.1800 |
| rs972283 | 7 | 130466854 | Primary hypertension | G/A | 0.0228 | 0.0000 | 3.80E-27 | 0.0024 | 0.0007 | 0.0003 |
| rs1011685 | 8 | 19830769 | Primary hypertension | C/T | 0.1162 | 0.0000 | 1.96E-277 | 0.0015 | 0.0011 | 0.1800 |
| rs2126259 | 8 | 9185146 | Primary hypertension | T/C | 0.0443 | 0.0000 | 5.99E-45 | 0.0042 | 0.0011 | 0.0001 |
| rs4738141 | 8 | 72469742 | Primary hypertension | G/A | 0.0174 | 0.0000 | 1.54E-12 | 0.0023 | 0.0008 | 0.0030 |
| rs7005992 | 8 | 126528955 | Primary hypertension | C/G | 0.0179 | 0.0000 | 2.02E-10 | 0.0009 | 0.0009 | 0.3200 |
| rs498313 | 9 | 78034169 | Primary hypertension | A/G | 0.0123 | 0.0000 | 3.51E-09 | -0.0007 | 0.0007 | 0.3100 |
| rs10995441 | 10 | 64869239 | Primary hypertension | G/T | 0.0163 | 0.0000 | 5.43E-12 | 0.0005 | 0.0008 | 0.5200 |
| rs11231693 | 11 | 63862612 | Primary hypertension | A/G | 0.0316 | 0.0000 | 4.68E-14 | 0.0061 | 0.0015 | 0.0000 |
| rs17402950 | 12 | 14571671 | Primary hypertension | G/A | 0.0312 | 0.0100 | 3.95E-08 | 0.0010 | 0.0013 | 0.4700 |
| rs718314 | 12 | 26453283 | Primary hypertension | G/A | 0.0161 | 0.0000 | 6.06E-13 | 0.0018 | 0.0008 | 0.0170 |
| rs7973683 | 12 | 124449223 | Primary hypertension | C/A | 0.0242 | 0.0000 | 2.91E-32 | 0.0005 | 0.0007 | 0.4700 |
| rs7323406 | 13 | 111628195 | Primary hypertension | A/G | 0.0150 | 0.0000 | 3.75E-07 | -0.0001 | 0.0007 | 0.9100 |
| rs7176058 | 15 | 39464167 | Primary hypertension | A/G | 0.0149 | 0.0000 | 8.20E-09 | 0.0009 | 0.0009 | 0.3100 |
| rs8032586 | 15 | 73081067 | Primary hypertension | C/T | 0.0213 | 0.0000 | 3.65E-07 | 0.0021 | 0.0011 | 0.0640 |
| rs754814 | 17 | 4657034 | Primary hypertension | T/C | 0.0117 | 0.0000 | 4.37E-08 | 0.0008 | 0.0008 | 0.3200 |
| rs7227237 | 18 | 47174679 | Primary hypertension | C/T | 0.0178 | 0.0000 | 1.60E-08 | 0.0012 | 0.0008 | 0.1300 |
| rs4804311 | 19 | 8615589 | Primary hypertension | A/G | 0.0367 | 0.0000 | 6.43E-25 | 0.0035 | 0.0012 | 0.0045 |
| rs4804833 | 19 | 7970635 | Primary hypertension | A/G | 0.0177 | 0.0000 | 4.03E-17 | 0.0020 | 0.0007 | 0.0029 |
| rs731839 | 19 | 33899065 | Primary hypertension | G/A | 0.0233 | 0.0000 | 3.23E-29 | 0.0009 | 0.0007 | 0.2200 |
| rs8101064 | 19 | 7293119 | Primary hypertension | T/C | 0.0565 | 0.0100 | 2.20E-12 | 0.0073 | 0.0019 | 0.0001 |
| rs6066149 | 20 | 45602638 | Primary hypertension | G/A | 0.0138 | 0.0000 | 5.34E-10 | 0.0008 | 0.0007 | 0.2700 |
| rs132985 | 22 | 38563471 | Primary hypertension | C/T | 0.0176 | 0.0000 | 6.70E-20 | -0.0001 | 0.0007 | 0.9400 |
| rs11577194 | 1 | 110500175 | Atrial fibrillation | T/C | 0.0135 | 0.0000 | 2.91E-12 | -0.0038 | 0.0067 | 0.5704 |
| rs17386142 | 1 | 50815783 | Atrial fibrillation | C/T | 0.0224 | 0.0000 | 1.10E-08 | 0.0301 | 0.0137 | 0.0274 |
| rs4846565 | 1 | 219722104 | Atrial fibrillation | G/A | 0.0163 | 0.0000 | 2.74E-15 | -0.0015 | 0.0070 | 0.8292 |
| rs683135 | 1 | 39895460 | Atrial fibrillation | A/G | 0.0191 | 0.0000 | 5.55E-19 | 0.0031 | 0.0072 | 0.6720 |
| rs9425291 | 1 | 172312769 | Atrial fibrillation | A/G | 0.0149 | 0.0000 | 3.43E-14 | -0.0035 | 0.0067 | 0.6023 |
| rs10195252 | 2 | 165513091 | Atrial fibrillation | T/C | 0.0272 | 0.0000 | 2.54E-40 | -0.0027 | 0.0067 | 0.6839 |
| rs2249105 | 2 | 65287896 | Atrial fibrillation | A/G | 0.0159 | 0.0000 | 1.21E-15 | 0.0624 | 0.0069 | 0.0000 |
| rs2943645 | 2 | 227099180 | Atrial fibrillation | T/C | 0.0306 | 0.0000 | 2.02E-52 | -0.0114 | 0.0069 | 0.0982 |
| rs492400 | 2 | 219349752 | Atrial fibrillation | T/C | 0.0130 | 0.0000 | 1.01E-10 | -0.0026 | 0.0067 | 0.6995 |
| rs11130329 | 3 | 52896855 | Atrial fibrillation | A/C | 0.0228 | 0.0000 | 5.64E-09 | -0.0212 | 0.0095 | 0.0257 |
| rs295449 | 3 | 47375955 | Atrial fibrillation | A/G | 0.0144 | 0.0000 | 1.92E-10 | -0.0112 | 0.0068 | 0.0975 |
| rs308971 | 3 | 12116620 | Atrial fibrillation | G/A | 0.0236 | 0.0000 | 2.64E-16 | -0.0208 | 0.0097 | 0.0314 |
| rs3864041 | 3 | 15185634 | Atrial fibrillation | T/C | 0.0109 | 0.0000 | 1.32E-07 | 0.0116 | 0.0068 | 0.0898 |
| rs645040 | 3 | 135926622 | Atrial fibrillation | T/G | 0.0250 | 0.0000 | 6.33E-26 | 0.0299 | 0.0079 | 0.0002 |
| rs9881942 | 3 | 123082416 | Atrial fibrillation | A/G | 0.0127 | 0.0000 | 5.90E-11 | 0.0067 | 0.0066 | 0.3160 |
| rs2699429 | 4 | 3480136 | Atrial fibrillation | C/T | 0.0165 | 0.0000 | 1.30E-15 | -0.0022 | 0.0069 | 0.7462 |
| rs3822072 | 4 | 89741269 | Atrial fibrillation | A/G | 0.0213 | 0.0000 | 7.72E-27 | -0.0120 | 0.0067 | 0.0744 |
| rs6822892 | 4 | 157734675 | Atrial fibrillation | A/G | 0.0182 | 0.0000 | 1.52E-18 | 0.0174 | 0.0077 | 0.0243 |
| rs1045241 | 5 | 118729286 | Atrial fibrillation | C/T | 0.0137 | 0.0000 | 4.62E-10 | -0.0031 | 0.0075 | 0.6795 |
| rs2434612 | 5 | 158022041 | Atrial fibrillation | G/A | 0.0168 | 0.0000 | 6.31E-12 | 0.0187 | 0.0081 | 0.0216 |
| rs459193 | 5 | 55806751 | Atrial fibrillation | G/A | 0.0222 | 0.0000 | 1.53E-23 | -0.0004 | 0.0075 | 0.9592 |
| rs4865796 | 5 | 53272664 | Atrial fibrillation | A/G | 0.0158 | 0.0000 | 4.30E-14 | -0.0099 | 0.0072 | 0.1672 |
| rs4976033 | 5 | 67714246 | Atrial fibrillation | G/A | 0.0169 | 0.0000 | 2.39E-15 | -0.0032 | 0.0069 | 0.6456 |
| rs6887914 | 5 | 112711486 | Atrial fibrillation | C/T | 0.0144 | 0.0000 | 2.86E-09 | 0.0111 | 0.0082 | 0.1761 |
| rs966544 | 5 | 173350405 | Atrial fibrillation | G/A | 0.0139 | 0.0000 | 3.74E-11 | -0.0475 | 0.0072 | 0.0000 |
| rs12525532 | 6 | 35004819 | Atrial fibrillation | T/C | 0.0149 | 0.0000 | 1.26E-13 | 0.0047 | 0.0069 | 0.4982 |
| rs2745353 | 6 | 127452935 | Atrial fibrillation | T/C | 0.0184 | 0.0000 | 5.78E-21 | -0.0042 | 0.0066 | 0.5250 |
| rs3861397 | 6 | 139828916 | Atrial fibrillation | G/A | 0.0206 | 0.0000 | 6.27E-24 | -0.0044 | 0.0067 | 0.5141 |
| rs6937438 | 6 | 43815364 | Atrial fibrillation | A/G | 0.0150 | 0.0000 | 4.37E-12 | -0.0022 | 0.0074 | 0.7633 |
| rs9492443 | 6 | 130398731 | Atrial fibrillation | C/T | 0.0144 | 0.0000 | 5.87E-11 | -0.0191 | 0.0076 | 0.0123 |
| rs17169104 | 7 | 15883727 | Atrial fibrillation | G/C | 0.0183 | 0.0000 | 1.23E-13 | 0.0025 | 0.0074 | 0.7304 |
| rs972283 | 7 | 130466854 | Atrial fibrillation | G/A | 0.0228 | 0.0000 | 3.80E-27 | -0.0118 | 0.0067 | 0.0763 |
| rs1011685 | 8 | 19830769 | Atrial fibrillation | C/T | 0.1162 | 0.0000 | 1.96E-277 | 0.0229 | 0.0112 | 0.0402 |
| rs2126259 | 8 | 9185146 | Atrial fibrillation | T/C | 0.0443 | 0.0000 | 5.99E-45 | -0.0160 | 0.0111 | 0.1487 |
| rs4738141 | 8 | 72469742 | Atrial fibrillation | G/A | 0.0174 | 0.0000 | 1.54E-12 | -0.0073 | 0.0076 | 0.3386 |
| rs7005992 | 8 | 126528955 | Atrial fibrillation | C/G | 0.0179 | 0.0000 | 2.02E-10 | 0.0066 | 0.0094 | 0.4824 |
| rs498313 | 9 | 78034169 | Atrial fibrillation | A/G | 0.0123 | 0.0000 | 3.51E-09 | -0.0071 | 0.0072 | 0.3263 |
| rs10995441 | 10 | 64869239 | Atrial fibrillation | G/T | 0.0163 | 0.0000 | 5.43E-12 | -0.0250 | 0.0081 | 0.0019 |
| rs11231693 | 11 | 63862612 | Atrial fibrillation | A/G | 0.0316 | 0.0000 | 4.68E-14 | -0.0170 | 0.0145 | 0.2406 |
| rs17402950 | 12 | 14571671 | Atrial fibrillation | G/A | 0.0312 | 0.0100 | 3.95E-08 | -0.0095 | 0.0139 | 0.4954 |
| rs718314 | 12 | 26453283 | Atrial fibrillation | G/A | 0.0161 | 0.0000 | 6.06E-13 | -0.0093 | 0.0076 | 0.2189 |
| rs7973683 | 12 | 124449223 | Atrial fibrillation | C/A | 0.0242 | 0.0000 | 2.91E-32 | 0.0345 | 0.0070 | 0.0000 |
| rs7323406 | 13 | 111628195 | Atrial fibrillation | A/G | 0.0150 | 0.0000 | 3.75E-07 | 0.0002 | 0.0073 | 0.9814 |
| rs7176058 | 15 | 39464167 | Atrial fibrillation | A/G | 0.0149 | 0.0000 | 8.20E-09 | 0.0140 | 0.0091 | 0.1263 |
| rs8032586 | 15 | 73081067 | Atrial fibrillation | C/T | 0.0213 | 0.0000 | 3.65E-07 | -0.0114 | 0.0114 | 0.3176 |
| rs754814 | 17 | 4657034 | Atrial fibrillation | T/C | 0.0117 | 0.0000 | 4.37E-08 | 0.0112 | 0.0074 | 0.1294 |
| rs7227237 | 18 | 47174679 | Atrial fibrillation | C/T | 0.0178 | 0.0000 | 1.60E-08 | -0.0020 | 0.0080 | 0.8067 |
| rs4804311 | 19 | 8615589 | Atrial fibrillation | A/G | 0.0367 | 0.0000 | 6.43E-25 | 0.0202 | 0.0125 | 0.1073 |
| rs4804833 | 19 | 7970635 | Atrial fibrillation | A/G | 0.0177 | 0.0000 | 4.03E-17 | 0.0083 | 0.0069 | 0.2291 |
| rs731839 | 19 | 33899065 | Atrial fibrillation | G/A | 0.0233 | 0.0000 | 3.23E-29 | -0.0100 | 0.0071 | 0.1569 |
| rs8101064 | 19 | 7293119 | Atrial fibrillation | T/C | 0.0565 | 0.0100 | 2.20E-12 | -0.0042 | 0.0195 | 0.8303 |
| rs6066149 | 20 | 45602638 | Atrial fibrillation | G/A | 0.0138 | 0.0000 | 5.34E-10 | 0.0065 | 0.0076 | 0.3942 |
| rs132985 | 22 | 38563471 | Atrial fibrillation | C/T | 0.0176 | 0.0000 | 6.70E-20 | -0.0134 | 0.0067 | 0.0443 |
| rs11577194 | 1 | 110500175 | Heart failure | T/C | 0.0135 | 0.0000 | 2.91E-12 | 0.0102 | 0.0078 | 0.1887 |
| rs17386142 | 1 | 50815783 | Heart failure | C/T | 0.0224 | 0.0000 | 1.10E-08 | 0.0083 | 0.0162 | 0.6078 |
| rs4846565 | 1 | 219722104 | Heart failure | G/A | 0.0163 | 0.0000 | 2.74E-15 | -0.0010 | 0.0082 | 0.9004 |
| rs683135 | 1 | 39895460 | Heart failure | A/G | 0.0191 | 0.0000 | 5.55E-19 | -0.0149 | 0.0085 | 0.0815 |
| rs9425291 | 1 | 172312769 | Heart failure | A/G | 0.0149 | 0.0000 | 3.43E-14 | 0.0221 | 0.0080 | 0.0057 |
| rs10195252 | 2 | 165513091 | Heart failure | T/C | 0.0272 | 0.0000 | 2.54E-40 | 0.0024 | 0.0080 | 0.7630 |
| rs2249105 | 2 | 65287896 | Heart failure | A/G | 0.0159 | 0.0000 | 1.21E-15 | 0.0039 | 0.0081 | 0.6306 |
| rs2943645 | 2 | 227099180 | Heart failure | T/C | 0.0306 | 0.0000 | 2.02E-52 | 0.0016 | 0.0082 | 0.8409 |
| rs492400 | 2 | 219349752 | Heart failure | T/C | 0.0130 | 0.0000 | 1.01E-10 | -0.0024 | 0.0079 | 0.7573 |
| rs11130329 | 3 | 52896855 | Heart failure | A/C | 0.0228 | 0.0000 | 5.64E-09 | -0.0164 | 0.0113 | 0.1458 |
| rs295449 | 3 | 47375955 | Heart failure | A/G | 0.0144 | 0.0000 | 1.92E-10 | -0.0033 | 0.0080 | 0.6784 |
| rs308971 | 3 | 12116620 | Heart failure | G/A | 0.0236 | 0.0000 | 2.64E-16 | 0.0074 | 0.0115 | 0.5201 |
| rs3864041 | 3 | 15185634 | Heart failure | T/C | 0.0109 | 0.0000 | 1.32E-07 | 0.0204 | 0.0081 | 0.0123 |
| rs645040 | 3 | 135926622 | Heart failure | T/G | 0.0250 | 0.0000 | 6.33E-26 | 0.0228 | 0.0097 | 0.0185 |
| rs9881942 | 3 | 123082416 | Heart failure | A/G | 0.0127 | 0.0000 | 5.90E-11 | -0.0154 | 0.0078 | 0.0482 |
| rs2699429 | 4 | 3480136 | Heart failure | C/T | 0.0165 | 0.0000 | 1.30E-15 | 0.0116 | 0.0081 | 0.1502 |
| rs3822072 | 4 | 89741269 | Heart failure | A/G | 0.0213 | 0.0000 | 7.72E-27 | 0.0044 | 0.0079 | 0.5759 |
| rs6822892 | 4 | 157734675 | Heart failure | A/G | 0.0182 | 0.0000 | 1.52E-18 | -0.0118 | 0.0098 | 0.2271 |
| rs1045241 | 5 | 118729286 | Heart failure | C/T | 0.0137 | 0.0000 | 4.62E-10 | 0.0152 | 0.0088 | 0.0859 |
| rs2434612 | 5 | 158022041 | Heart failure | G/A | 0.0168 | 0.0000 | 6.31E-12 | -0.0066 | 0.0097 | 0.4964 |
| rs459193 | 5 | 55806751 | Heart failure | G/A | 0.0222 | 0.0000 | 1.53E-23 | 0.0060 | 0.0090 | 0.5086 |
| rs4865796 | 5 | 53272664 | Heart failure | A/G | 0.0158 | 0.0000 | 4.30E-14 | -0.0147 | 0.0084 | 0.0817 |
| rs4976033 | 5 | 67714246 | Heart failure | G/A | 0.0169 | 0.0000 | 2.39E-15 | 0.0200 | 0.0082 | 0.0150 |
| rs6887914 | 5 | 112711486 | Heart failure | C/T | 0.0144 | 0.0000 | 2.86E-09 | -0.0020 | 0.0095 | 0.8344 |
| rs966544 | 5 | 173350405 | Heart failure | G/A | 0.0139 | 0.0000 | 3.74E-11 | -0.0280 | 0.0084 | 0.0009 |
| rs12525532 | 6 | 35004819 | Heart failure | T/C | 0.0149 | 0.0000 | 1.26E-13 | 0.0037 | 0.0082 | 0.6512 |
| rs2745353 | 6 | 127452935 | Heart failure | T/C | 0.0184 | 0.0000 | 5.78E-21 | -0.0122 | 0.0079 | 0.1215 |
| rs3861397 | 6 | 139828916 | Heart failure | G/A | 0.0206 | 0.0000 | 6.27E-24 | -0.0019 | 0.0082 | 0.8163 |
| rs6937438 | 6 | 43815364 | Heart failure | A/G | 0.0150 | 0.0000 | 4.37E-12 | -0.0026 | 0.0089 | 0.7670 |
| rs9492443 | 6 | 130398731 | Heart failure | C/T | 0.0144 | 0.0000 | 5.87E-11 | -0.0167 | 0.0089 | 0.0626 |
| rs17169104 | 7 | 15883727 | Heart failure | G/C | 0.0183 | 0.0000 | 1.23E-13 | 0.0048 | 0.0086 | 0.5748 |
| rs972283 | 7 | 130466854 | Heart failure | G/A | 0.0228 | 0.0000 | 3.80E-27 | 0.0258 | 0.0080 | 0.0012 |
| rs1011685 | 8 | 19830769 | Heart failure | C/T | 0.1162 | 0.0000 | 1.96E-277 | 0.0348 | 0.0134 | 0.0093 |
| rs2126259 | 8 | 9185146 | Heart failure | T/C | 0.0443 | 0.0000 | 5.99E-45 | -0.0059 | 0.0135 | 0.6633 |
| rs4738141 | 8 | 72469742 | Heart failure | G/A | 0.0174 | 0.0000 | 1.54E-12 | 0.0012 | 0.0091 | 0.8936 |
| rs7005992 | 8 | 126528955 | Heart failure | C/G | 0.0179 | 0.0000 | 2.02E-10 | 0.0075 | 0.0113 | 0.5039 |
| rs498313 | 9 | 78034169 | Heart failure | A/G | 0.0123 | 0.0000 | 3.51E-09 | -0.0126 | 0.0085 | 0.1380 |
| rs10995441 | 10 | 64869239 | Heart failure | G/T | 0.0163 | 0.0000 | 5.43E-12 | -0.0092 | 0.0096 | 0.3359 |
| rs11231693 | 11 | 63862612 | Heart failure | A/G | 0.0316 | 0.0000 | 4.68E-14 | -0.0071 | 0.0178 | 0.6914 |
| rs17402950 | 12 | 14571671 | Heart failure | G/A | 0.0312 | 0.0100 | 3.95E-08 | 0.0162 | 0.0159 | 0.3083 |
| rs718314 | 12 | 26453283 | Heart failure | G/A | 0.0161 | 0.0000 | 6.06E-13 | -0.0082 | 0.0090 | 0.3608 |
| rs7973683 | 12 | 124449223 | Heart failure | C/A | 0.0242 | 0.0000 | 2.91E-32 | 0.0187 | 0.0083 | 0.0239 |
| rs7323406 | 13 | 111628195 | Heart failure | A/G | 0.0150 | 0.0000 | 3.75E-07 | 0.0079 | 0.0087 | 0.3647 |
| rs7176058 | 15 | 39464167 | Heart failure | A/G | 0.0149 | 0.0000 | 8.20E-09 | 0.0178 | 0.0109 | 0.1030 |
| rs8032586 | 15 | 73081067 | Heart failure | C/T | 0.0213 | 0.0000 | 3.65E-07 | 0.0035 | 0.0134 | 0.7958 |
| rs754814 | 17 | 4657034 | Heart failure | T/C | 0.0117 | 0.0000 | 4.37E-08 | 0.0206 | 0.0088 | 0.0195 |
| rs7227237 | 18 | 47174679 | Heart failure | C/T | 0.0178 | 0.0000 | 1.60E-08 | 0.0160 | 0.0095 | 0.0927 |
| rs4804311 | 19 | 8615589 | Heart failure | A/G | 0.0367 | 0.0000 | 6.43E-25 | 0.0494 | 0.0145 | 0.0006 |
| rs4804833 | 19 | 7970635 | Heart failure | A/G | 0.0177 | 0.0000 | 4.03E-17 | 0.0121 | 0.0082 | 0.1383 |
| rs731839 | 19 | 33899065 | Heart failure | G/A | 0.0233 | 0.0000 | 3.23E-29 | -0.0044 | 0.0084 | 0.6052 |
| rs8101064 | 19 | 7293119 | Heart failure | T/C | 0.0565 | 0.0100 | 2.20E-12 | -0.0033 | 0.0238 | 0.8898 |
| rs6066149 | 20 | 45602638 | Heart failure | G/A | 0.0138 | 0.0000 | 5.34E-10 | 0.0001 | 0.0088 | 0.9928 |
| rs132985 | 22 | 38563471 | Heart failure | C/T | 0.0176 | 0.0000 | 6.70E-20 | -0.0226 | 0.0079 | 0.0042 |
| rs11577194 | 1 | 109957553 | PAD | T/C | 0.0135 | 0.0000 | 2.91E-12 | 0.0210 | 0.0429 | 0.6249 |
| rs17386142 | 1 | 50350111 | PAD | C/T | 0.0224 | 0.0000 | 1.10E-08 | 0.0217 | 0.1053 | 0.8365 |
| rs4846565 | 1 | 219548762 | PAD | G/A | 0.0163 | 0.0000 | 2.74E-15 | 0.0125 | 0.0446 | 0.7790 |
| rs683135 | 1 | 39429788 | PAD | A/G | 0.0191 | 0.0000 | 5.55E-19 | 0.0599 | 0.0492 | 0.2234 |
| rs9425291 | 1 | 172343629 | PAD | A/G | 0.0149 | 0.0000 | 3.43E-14 | 0.0512 | 0.0430 | 0.2333 |
| rs10195252 | 2 | 164656581 | PAD | T/C | 0.0272 | 0.0000 | 2.54E-40 | 0.0341 | 0.0446 | 0.4449 |
| rs2249105 | 2 | 65060762 | PAD | A/G | 0.0159 | 0.0000 | 1.21E-15 | -0.0605 | 0.0454 | 0.1831 |
| rs2943645 | 2 | 226234464 | PAD | T/C | 0.0306 | 0.0000 | 2.02E-52 | 0.0153 | 0.0444 | 0.7305 |
| rs492400 | 2 | 218485029 | PAD | T/C | 0.0130 | 0.0000 | 1.01E-10 | 0.0294 | 0.0431 | 0.4953 |
| rs11130329 | 3 | 52862839 | PAD | A/C | 0.0228 | 0.0000 | 5.64E-09 | -0.0003 | 0.0718 | 0.9962 |
| rs295449 | 3 | 47334465 | PAD | A/G | 0.0144 | 0.0000 | 1.92E-10 | -0.0208 | 0.0438 | 0.6358 |
| rs308971 | 3 | 12075120 | PAD | G/A | 0.0236 | 0.0000 | 2.64E-16 | 0.0324 | 0.0703 | 0.6447 |
| rs3864041 | 3 | 15144127 | PAD | T/C | 0.0109 | 0.0000 | 1.32E-07 | -0.0819 | 0.0457 | 0.0731 |
| rs645040 | 3 | 136207780 | PAD | T/G | 0.0250 | 0.0000 | 6.33E-26 | -0.0718 | 0.0608 | 0.2376 |
| rs9881942 | 3 | 123363569 | PAD | A/G | 0.0127 | 0.0000 | 5.90E-11 | -0.0968 | 0.0429 | 0.0239 |
| rs2699429 | 4 | 3478409 | PAD | C/T | 0.0165 | 0.0000 | 1.30E-15 | 0.0085 | 0.0454 | 0.8512 |
| rs3822072 | 4 | 88820118 | PAD | A/G | 0.0213 | 0.0000 | 7.72E-27 | 0.0781 | 0.0427 | 0.0675 |
| rs6822892 | 4 | 156813523 | PAD | A/G | 0.0182 | 0.0000 | 1.52E-18 | -0.0018 | 0.0493 | 0.9711 |
| rs1045241 | 5 | 119393591 | PAD | C/T | 0.0137 | 0.0000 | 4.62E-10 | -0.1000 | 0.0459 | 0.0295 |
| rs2434612 | 5 | 158595033 | PAD | G/A | 0.0168 | 0.0000 | 6.31E-12 | -0.0320 | 0.0642 | 0.6183 |
| rs459193 | 5 | 56510924 | PAD | G/A | 0.0222 | 0.0000 | 1.53E-23 | -0.0304 | 0.0458 | 0.5063 |
| rs4865796 | 5 | 53976834 | PAD | A/G | 0.0158 | 0.0000 | 4.30E-14 | -0.0329 | 0.0451 | 0.4655 |
| rs4976033 | 5 | 68418419 | PAD | G/A | 0.0169 | 0.0000 | 2.39E-15 | -0.0534 | 0.0437 | 0.2213 |
| rs6887914 | 5 | 113375789 | PAD | C/T | 0.0144 | 0.0000 | 2.86E-09 | -0.0896 | 0.0561 | 0.1103 |
| rs966544 | 5 | 173923402 | PAD | G/A | 0.0139 | 0.0000 | 3.74E-11 | -0.0069 | 0.0433 | 0.8727 |
| rs12525532 | 6 | 35037042 | PAD | T/C | 0.0149 | 0.0000 | 1.26E-13 | -0.0372 | 0.0432 | 0.3892 |
| rs2745353 | 6 | 127131790 | PAD | T/C | 0.0184 | 0.0000 | 5.78E-21 | -0.0411 | 0.0428 | 0.3375 |
| rs3861397 | 6 | 139507779 | PAD | G/A | 0.0206 | 0.0000 | 6.27E-24 | -0.0398 | 0.0433 | 0.3581 |
| rs6937438 | 6 | 43847627 | PAD | A/G | 0.0150 | 0.0000 | 4.37E-12 | 0.0195 | 0.0460 | 0.6717 |
| rs9492443 | 6 | 130077586 | PAD | C/T | 0.0144 | 0.0000 | 5.87E-11 | -0.0439 | 0.0487 | 0.3681 |
| rs17169104 | 7 | 15844102 | PAD | G/C | 0.0183 | 0.0000 | 1.23E-13 | 0.0753 | 0.0473 | 0.1115 |
| rs972283 | 7 | 130782095 | PAD | G/A | 0.0228 | 0.0000 | 3.80E-27 | 0.0304 | 0.0428 | 0.4773 |
| rs1011685 | 8 | 19973258 | PAD | C/T | 0.1162 | 0.0000 | 1.96E-277 | 0.0070 | 0.0753 | 0.9265 |
| rs2126259 | 8 | 9327636 | PAD | T/C | 0.0443 | 0.0000 | 5.99E-45 | -0.0117 | 0.0583 | 0.8412 |
| rs4738141 | 8 | 71557507 | PAD | G/A | 0.0174 | 0.0000 | 1.54E-12 | -0.1238 | 0.0492 | 0.0119 |
| rs7005992 | 8 | 125516713 | PAD | C/G | 0.0179 | 0.0000 | 2.02E-10 | -0.1473 | 0.0704 | 0.0363 |
| rs498313 | 9 | 75419253 | PAD | A/G | 0.0123 | 0.0000 | 3.51E-09 | 0.0285 | 0.0491 | 0.5625 |
| rs10995441 | 10 | 63109479 | PAD | G/T | 0.0163 | 0.0000 | 5.43E-12 | 0.0120 | 0.0543 | 0.8246 |
| rs11231693 | 11 | 64095140 | PAD | A/G | 0.0316 | 0.0000 | 4.68E-14 | 0.1814 | 0.0800 | 0.0234 |
| rs17402950 | 12 | 14418737 | PAD | G/A | 0.0312 | 0.0100 | 3.95E-08 | 0.0034 | 0.0852 | 0.9681 |
| rs718314 | 12 | 26300350 | PAD | G/A | 0.0161 | 0.0000 | 6.06E-13 | 0.0599 | 0.0472 | 0.2051 |
| rs7973683 | 12 | 123964676 | PAD | C/A | 0.0242 | 0.0000 | 2.91E-32 | -0.0570 | 0.0467 | 0.2226 |
| rs7323406 | 13 | 110975848 | PAD | A/G | 0.0150 | 0.0000 | 3.75E-07 | -0.0822 | 0.0485 | 0.0902 |
| rs7176058 | 15 | 39171966 | PAD | A/G | 0.0149 | 0.0000 | 8.20E-09 | -0.0453 | 0.0545 | 0.4061 |
| rs8032586 | 15 | 72788726 | PAD | C/T | 0.0213 | 0.0000 | 3.65E-07 | 0.0841 | 0.0557 | 0.1309 |
| rs754814 | 17 | 4753739 | PAD | T/C | 0.0117 | 0.0000 | 4.37E-08 | -0.0045 | 0.0495 | 0.9280 |
| rs7227237 | 18 | 49648309 | PAD | C/T | 0.0178 | 0.0000 | 1.60E-08 | 0.0330 | 0.0475 | 0.4876 |
| rs4804311 | 19 | 8550705 | PAD | A/G | 0.0367 | 0.0000 | 6.43E-25 | -0.0429 | 0.0838 | 0.6084 |
| rs4804833 | 19 | 7905750 | PAD | A/G | 0.0177 | 0.0000 | 4.03E-17 | 0.0035 | 0.0441 | 0.9359 |
| rs731839 | 19 | 33408159 | PAD | G/A | 0.0233 | 0.0000 | 3.23E-29 | 0.0367 | 0.0448 | 0.4134 |
| rs8101064 | 19 | 7293108 | PAD | T/C | 0.0565 | 0.0100 | 2.20E-12 | 0.1386 | 0.0888 | 0.1184 |
| rs6066149 | 20 | 46973999 | PAD | G/A | 0.0138 | 0.0000 | 5.34E-10 | 0.0136 | 0.0522 | 0.7939 |
| rs132985 | 22 | 38167464 | PAD | C/T | 0.0176 | 0.0000 | 6.70E-20 | -0.0576 | 0.0432 | 0.1826 |
| rs11577194 | 1 | 110500175 | VTE | T/C | 0.0135 | 0.0000 | 2.91E-12 | 0.0001 | 0.0003 | 0.6310 |
| rs17386142 | 1 | 50815783 | VTE | C/T | 0.0224 | 0.0000 | 1.10E-08 | -0.0010 | 0.0005 | 0.0361 |
| rs4846565 | 1 | 219722104 | VTE | G/A | 0.0163 | 0.0000 | 2.74E-15 | 0.0000 | 0.0003 | 0.9174 |
| rs683135 | 1 | 39895460 | VTE | A/G | 0.0191 | 0.0000 | 5.55E-19 | 0.0003 | 0.0003 | 0.2472 |
| rs9425291 | 1 | 172312769 | VTE | A/G | 0.0149 | 0.0000 | 3.43E-14 | 0.0001 | 0.0003 | 0.6951 |
| rs10195252 | 2 | 165513091 | VTE | T/C | 0.0272 | 0.0000 | 2.54E-40 | -0.0005 | 0.0003 | 0.0455 |
| rs2249105 | 2 | 65287896 | VTE | A/G | 0.0159 | 0.0000 | 1.21E-15 | -0.0003 | 0.0003 | 0.3070 |
| rs2943645 | 2 | 227099180 | VTE | T/C | 0.0306 | 0.0000 | 2.02E-52 | 0.0004 | 0.0003 | 0.1815 |
| rs492400 | 2 | 219349752 | VTE | T/C | 0.0130 | 0.0000 | 1.01E-10 | 0.0001 | 0.0003 | 0.8380 |
| rs11130329 | 3 | 52896855 | VTE | A/C | 0.0228 | 0.0000 | 5.64E-09 | -0.0003 | 0.0004 | 0.3928 |
| rs295449 | 3 | 47375955 | VTE | A/G | 0.0144 | 0.0000 | 1.92E-10 | 0.0003 | 0.0003 | 0.2114 |
| rs308971 | 3 | 12116620 | VTE | G/A | 0.0236 | 0.0000 | 2.64E-16 | -0.0003 | 0.0004 | 0.3920 |
| rs3864041 | 3 | 15185634 | VTE | T/C | 0.0109 | 0.0000 | 1.32E-07 | 0.0004 | 0.0003 | 0.1695 |
| rs645040 | 3 | 135926622 | VTE | T/G | 0.0250 | 0.0000 | 6.33E-26 | 0.0004 | 0.0003 | 0.2477 |
| rs9881942 | 3 | 123082416 | VTE | A/G | 0.0127 | 0.0000 | 5.90E-11 | -0.0004 | 0.0003 | 0.1061 |
| rs2699429 | 4 | 3480136 | VTE | C/T | 0.0165 | 0.0000 | 1.30E-15 | -0.0002 | 0.0003 | 0.3684 |
| rs3822072 | 4 | 89741269 | VTE | A/G | 0.0213 | 0.0000 | 7.72E-27 | 0.0002 | 0.0003 | 0.3687 |
| rs6822892 | 4 | 157734675 | VTE | A/G | 0.0182 | 0.0000 | 1.52E-18 | -0.0001 | 0.0003 | 0.7783 |
| rs1045241 | 5 | 118729286 | VTE | C/T | 0.0137 | 0.0000 | 4.62E-10 | 0.0005 | 0.0003 | 0.0770 |
| rs2434612 | 5 | 158022041 | VTE | G/A | 0.0168 | 0.0000 | 6.31E-12 | -0.0004 | 0.0003 | 0.2393 |
| rs459193 | 5 | 55806751 | VTE | G/A | 0.0222 | 0.0000 | 1.53E-23 | 0.0001 | 0.0003 | 0.6503 |
| rs4865796 | 5 | 53272664 | VTE | A/G | 0.0158 | 0.0000 | 4.30E-14 | 0.0000 | 0.0003 | 0.9410 |
| rs4976033 | 5 | 67714246 | VTE | G/A | 0.0169 | 0.0000 | 2.39E-15 | 0.0002 | 0.0003 | 0.4860 |
| rs6887914 | 5 | 112711486 | VTE | C/T | 0.0144 | 0.0000 | 2.86E-09 | -0.0001 | 0.0003 | 0.8137 |
| rs966544 | 5 | 173350405 | VTE | G/A | 0.0139 | 0.0000 | 3.74E-11 | -0.0008 | 0.0003 | 0.0047 |
| rs12525532 | 6 | 35004819 | VTE | T/C | 0.0149 | 0.0000 | 1.26E-13 | 0.0008 | 0.0003 | 0.0023 |
| rs2745353 | 6 | 127452935 | VTE | T/C | 0.0184 | 0.0000 | 5.78E-21 | 0.0002 | 0.0003 | 0.4240 |
| rs3861397 | 6 | 139828916 | VTE | G/A | 0.0206 | 0.0000 | 6.27E-24 | -0.0002 | 0.0003 | 0.5139 |
| rs6937438 | 6 | 43815364 | VTE | A/G | 0.0150 | 0.0000 | 4.37E-12 | 0.0002 | 0.0003 | 0.4893 |
| rs9492443 | 6 | 130398731 | VTE | C/T | 0.0144 | 0.0000 | 5.87E-11 | 0.0001 | 0.0003 | 0.6950 |
| rs17169104 | 7 | 15883727 | VTE | G/C | 0.0183 | 0.0000 | 1.23E-13 | -0.0006 | 0.0003 | 0.0594 |
| rs972283 | 7 | 130466854 | VTE | G/A | 0.0228 | 0.0000 | 3.80E-27 | 0.0000 | 0.0003 | 0.9213 |
| rs1011685 | 8 | 19830769 | VTE | C/T | 0.1162 | 0.0000 | 1.96E-277 | -0.0009 | 0.0004 | 0.0386 |
| rs2126259 | 8 | 9185146 | VTE | T/C | 0.0443 | 0.0000 | 5.99E-45 | 0.0007 | 0.0004 | 0.0871 |
| rs4738141 | 8 | 72469742 | VTE | G/A | 0.0174 | 0.0000 | 1.54E-12 | -0.0002 | 0.0003 | 0.4415 |
| rs7005992 | 8 | 126528955 | VTE | C/G | 0.0179 | 0.0000 | 2.02E-10 | -0.0003 | 0.0004 | 0.4500 |
| rs498313 | 9 | 78034169 | VTE | A/G | 0.0123 | 0.0000 | 3.51E-09 | -0.0002 | 0.0003 | 0.5264 |
| rs10995441 | 10 | 64869239 | VTE | G/T | 0.0163 | 0.0000 | 5.43E-12 | 0.0004 | 0.0003 | 0.2334 |
| rs11231693 | 11 | 63862612 | VTE | A/G | 0.0316 | 0.0000 | 4.68E-14 | -0.0007 | 0.0006 | 0.2073 |
| rs17402950 | 12 | 14571671 | VTE | G/A | 0.0312 | 0.0100 | 3.95E-08 | 0.0001 | 0.0005 | 0.8832 |
| rs718314 | 12 | 26453283 | VTE | G/A | 0.0161 | 0.0000 | 6.06E-13 | 0.0000 | 0.0003 | 0.9190 |
| rs7973683 | 12 | 124449223 | VTE | C/A | 0.0242 | 0.0000 | 2.91E-32 | -0.0001 | 0.0003 | 0.7163 |
| rs7323406 | 13 | 111628195 | VTE | A/G | 0.0150 | 0.0000 | 3.75E-07 | 0.0006 | 0.0003 | 0.0440 |
| rs7176058 | 15 | 39464167 | VTE | A/G | 0.0149 | 0.0000 | 8.20E-09 | 0.0001 | 0.0004 | 0.8572 |
| rs8032586 | 15 | 73081067 | VTE | C/T | 0.0213 | 0.0000 | 3.65E-07 | 0.0008 | 0.0005 | 0.0740 |
| rs754814 | 17 | 4657034 | VTE | T/C | 0.0117 | 0.0000 | 4.37E-08 | 0.0000 | 0.0003 | 0.9670 |
| rs7227237 | 18 | 47174679 | VTE | C/T | 0.0178 | 0.0000 | 1.60E-08 | -0.0002 | 0.0003 | 0.6362 |
| rs4804311 | 19 | 8615589 | VTE | A/G | 0.0367 | 0.0000 | 6.43E-25 | 0.0007 | 0.0005 | 0.1268 |
| rs4804833 | 19 | 7970635 | VTE | A/G | 0.0177 | 0.0000 | 4.03E-17 | -0.0003 | 0.0003 | 0.2429 |
| rs731839 | 19 | 33899065 | VTE | G/A | 0.0233 | 0.0000 | 3.23E-29 | -0.0007 | 0.0003 | 0.0123 |
| rs8101064 | 19 | 7293119 | VTE | T/C | 0.0565 | 0.0100 | 2.20E-12 | 0.0002 | 0.0008 | 0.7947 |
| rs6066149 | 20 | 45602638 | VTE | G/A | 0.0138 | 0.0000 | 5.34E-10 | 0.0003 | 0.0003 | 0.2945 |
| rs132985 | 22 | 38563471 | VTE | C/T | 0.0176 | 0.0000 | 6.70E-20 | -0.0008 | 0.0003 | 0.0034 |

Chr, Chromosome; CVD, cardiovascular disease; EA, effect allele; OA, other allele; Pos, position; SE, standard error; SNP, single nucleotide polymorphism; The unit of beta coefficients is log-odds per allele.

**Supplemental Table S8:** Inverse-variance weighted MR estimates between insulin resistance and cardiovascular diseases, after excluding SNPs that are nominally associated with BMI.

| **Outcome** | **SΝPs** | **OR** | **95% CI** | **P-value** | **P-value for heterogeneity** |
| --- | --- | --- | --- | --- | --- |
| Hypertension | 44 | 1.056 | 1.038-1.074 | **7.48E-10** | **3.76×10^-9^** |
| Atrial fibrillation | 44 | 1.013 | 0.819-1.253 | 0.904 | **6.82×10^-17^** |
| Heart failure | 44 | 1.235 | 1.034-1.475 | ***0.020*** | **6.23×10^-5^** |
| PAD | 44 | 1.817 | 1.315-2.509 | **2.87E-04** | 0.530 |
| VTE | 44 | 0.998 | 0.993-1.004 | 0.568 | **0.005** |
| Aortic aneurysm | 44 | 1.671 | 0.927-3.011 | 0.087 | 0.125 |
| **Abbreviations:** CI, Confidence Interval; MR, mendelian randomization; OR, Odds Ratio; PAD, peripheral artery disease; SNPs, single nucleotide polymorphisms; VTE, venous thromboembolism. | | | | | |

**Supplemental Table S9:** Sensitivity analyses of the Mendelian randomization study on insulin resistance and cardiovascular disease.

|  |  | **Sensitivity analyses** | | | | | | | | | |
| --- | --- | --- | --- | --- | --- | --- | --- | --- | --- | --- | --- |
|  |  | **Maximum likelihood** | | **Weighted median** | | **MR-Egger** | | | **MR-PRESSO** | | |
| **Outcome** | **N** | OR (95% CI) | P-value | OR (95% CI) | P-value | OR (95% CI) | P-value | Intercept (95% CI) | SNPs | OR (95% CI) | P-value |
| **Hypertension** |  |  |  |  |  |  |  |  |  |  |  |
| 53-SNPs | 54,358 | 1.06 (1.05-1.07) | **1.29E-31** | 1.04 (1.02-1.06) | **9.50E-06** | 1.04 (1.00-1.07) | ***4.38E-02*** | 0.001 (0.000; 0.001) | 50 | 1.08 (1.06-1.09) | **1.83E-11** |
| 52-SNPs | 54,358 | 1.08 (1.06-1.09) | **2.95E-38** | 1.08 (1.06-1.10) | **2.87E-16** | 1.12 (1.06-1.19) | **1.86E-04** | -0.001 (-0.002;0.000) | 49 | 1.08 (1.06-1.09) | **1.13E-12** |
| 44-SNPs | 54,358 | 1.06 (1.05-1.07) | **2.62E-24** | 1.03 (1.02-1.05) | **2.77E-04** | 1.02 (0.99-1.05) | 1.85E-01 | 0.001 (0.000; 0.002) | 41 | 1.08 (1.06-1.09) | **6.48E-11** |
| 28-SNPs | 54,358 | 1.07 (1.05-1.09) | **7.83E-13** | 1.07 (1.04-1.10) | **1.33E-05** | 1.19 (1.06-1.34) | ***8.45E-03*** | -0.002 (-0.004; 0.000) | 27 | 1.08 (1.05-1.10) | **5.34E-06** |
| **Atrial fibrillation** |  |  |  |  |  |  |  |  |  |  |  |
| 53-SNPs | 60,620 | 1.02 (0.93-1.13) | 0.637 | 0.94 (0.80-1.11) | 0.469 | 1.15 (0.75-1.76) | 0.528 | -0.003 (-0.012; 0.006) | 48 | 0.95 (0.83-1.08) | 0.419 |
| 52-SNPs | 60,620 | 0.96 (0.86-1.08) | 0.505 | 0.81 (0.69-0.96) | ***0.018*** | 0.89 (0.40-1.98) | 0.773 | 0.002 (-0.014; 0.017) | 47 | 0.86 (0.75-1.00) | 0.051 |
| 44-SNPs | 60,620 | 1.01 (0.91-1.13) | 0.803 | 0.99 (0.84-1.18) | 0.948 | 1.08 (0.72-1.62) | 0.700 | -0.002 (-0.011; 0.007) | 42 | 0.98 (0.85-1.13) | 0.750 |
| 28-SNPs | 60,620 | 1.09 (0.90-1.33) | 0.384 | 0.82 (0.62-1.10) | 0.180 | 0.69 (0.08-6.03) | 0.739 | 0.007 (-0.026;0.040) | 26 | 1.02 (0.79-1.31) | 0.900 |
| **Heart failure** |  |  |  |  |  |  |  |  |  |  |  |
| 53-SNPs | 47,309 | 1.20 (1.07-1.34) | **0.002** | 1.31 (1.08-1.59) | **0.006** | 1.40 (1.00-1.98) | 0.059 | -0.004 (-0.012; 0.003) | 51 | 1.25 (1.07-1.46) | **0.008** |
| 52-SNPs | 47,309 | 1.15 (1.01-1.31) | ***0.036*** | 1.07 (0.87-1.33) | 0.501 | 1.42 (0.74-2.73) | 0.296 | -0.004 (-0.017; 0.008) | 51 | 1.18 (0.97-1.42) | 0.097 |
| 44-SNPs | 47,309 | 1.24 (1.09-1.41) | ***0.001*** | 1.32 (1.08-1.62) | **0.008** | 1.30 (0.92-1.84) | 0.139 | -0.001 (-0.009; 0.006) | 44 | 1.24 (1.03-1.48) | ***0.024*** |
| 28-SNPs | 47,309 | 1.17 (0.94-1.45) | 0.167 | 1.25 (0.90-1.73) | 0.190 | 1.10 (0.26-4.74) | 0.897 | 0.001 (-0.022; 0.024) | 27 | 1.25 (0.94-1.66) | 0.142 |
| **PAD** |  |  |  |  |  |  |  |  |  |  |  |
| 53-SNPs | 5323 | 1.90(1.411-2.58) | **2.38E-05** | 1.91 (1.18-3.10) | ***0.009*** | 2.28 (1.25-4.18) | ***0.010*** | -0.005 (-0.018; 0.008) | 53 | 1.90 (1.43-2.54) | **5.78E-05** |
| 52-SNPs | 5323 | 1.97 (1.40-2.77) | **9.85E-05** | 2.21 (1.36-3.58) | **0.001** | 4.07 (1.44-11.51) | ***0.011*** | -0.015 (-0.035; 0.004) | 52 | 1.96 (1.41-2.73) | **2.03E-04** |
| 44-SNPs | 5323 | 1.83 (1.32-2.55) | **3.30E-04** | 1.84 (1.08-3.13) | **0.026** | 2.05 (1.09-3.86) | ***0.031*** | -0.003 (-0.017; 0.011) | 44 | 1.82 (1.32-2.51) | **7.56E-04** |
| 28-SNPs | 5323 | 2.04 (1.15-3.64) | ***0.015*** | 1.80 (0.79-4.11) | 0.161 | 10.12 (0.77-133.23) | 0.090 | -0.026 (-0.067; 0.015) | 28 | 2.02 (1.12-3.65) | ***0.026*** |
| **VTE** |  |  |  |  |  |  |  |  |  |  |  |
| 53-SNPs | 4620 | 1.00 (0.99-1.00) | 0.238 | 0.99 (0.99-1.00) | 0.071 | 0.99 (0.98-1.00) | 0.216 | 0.000 (0.000; 0.000) | 53 | 1.00 (0.99-1.00) | 0.372 |
| 52-SNPs | 4620 | 1.00 (1.00-1.00) | 0.873 | 1.00 (1.00-1.01) | 0.574 | 1.00 (0.98-1.02) | 0.902 | 0.000 (0.000; 0.000) | 52 | 1.00 (0.99-1.01) | 0.903 |
| 44-SNPs | 4620 | 1.00 (0.99-1.00) | 0.466 | 0.99 (0.99-1.00) | 0.059 | 0.99 (0.98-1.00) | 0.124 | 0.000 (0.000; 0.000) | 44 | 1.00 (0.99-1.00) | 0.570 |
| 28-SNPs | 4620 | 1.00 (0.99-1.01) | 0.737 | 1.00 (0.99-1.01) | 0.768 | 0.97 (0.93-1.01) | 0.207 | 0.000 (0.000; 0.001) | 28 | 1.00 (0.99-1.01) | 0.801 |
| **Aortic aneurysm** |  |  |  |  |  |  |  |  |  |  |  |
| 53-SNPs | 1919 | 1.57 (0.97-2.54) | 0.067 | 1.19 (0.52-2.76) | 0.681 | 1.03 (0.36-2.98) | 0.954 | 0.011 (-0.013; 0.034) | 53 | 1.57 (0.93; 2.65) | 0.094 |
| 52-SNPs | 1919 | 1.76 (1.02-3.06) | ***0.043*** | 1.63 (0.70-3.78) | 0.256 | 1.03 (0.16-6.51) | 0.976 | 0.011 (-0.025; 0.047) | 52 | 1.73 (0.93; 2.65) | 0.076 |
| 44-SNPs | 1919 | 1.71 (1.00-2.90) | ***0.049*** | 1.18 (0.49-2.83) | 0.706 | 1.11 (0.36-3.47) | 0.855 | 0.011 (-0.015; 0.036) | 44 | 1.67 (0.93-3.01) | 0.094 |
| 28-SNPs | 1919 | 1.37 (0.54-3.46) | 0.505 | 1.50 (0.37-6.07) | 0.570 | 0.73 (0.01-58.66) | 0.889 | 0.010 (-0.060; 0.080) | 28 | 1.36 (0.51; 3.61) | 0.542 |

P-values below the Bonferroni-corrected threshold of 8.33×10^-3^ are displayed in bold and suggestive P-values between 0.05 and 8.33×10^-3^ are displayed in bold-italic. Odds ratios represent the associations of 1-SD change in insulin resistance with the risk of cardiovascular diseases.

**Abbreviations:** MR, mendelian randomization; MR-PRESSO, MR pleiotropy residual sum and outlier; N, the number of cases included in each sensitivity analyses; PAD, peripheral artery disease; VTE, venous thromboembolism.

**Supplemental Table S10:** Multivariable Mendelian randomization associations of insulin resistance with CVD risk adjusting for lipid traits.

| **Exposure** | **SNPs** | **OR** | **95% CI** | **P-value** |
| --- | --- | --- | --- | --- |
| **Hypertension** | | | | |
| IR | 66 | 1.02 | 1.00-1.04 | ***0.022*** |
| HDL-C | 91 | 1.01 | 1.01-1.02 | **7.58E-04** |
| TG | 55 | 1.01 | 1.00-1.02 | **8.77E-03** |
| **Heart failure** | | | | |
| IR | 64 | 0.96 | 0.80-1.14 | 0.616 |
| HDL-C | 91 | 1.02 | 0.95-1.11 | 0.533 |
| TG | 55 | 1.13 | 1.04-1.23 | **4.90E-03** |
| **PAD** | | | | |
| IR | 55 | 0.75 | 0.42-1.36 | 0.347 |
| HDL-C | 80 | 1.09 | 0.84-1.43 | 0.517 |
| TG | 47 | 1.12 | 0.84-1.48 | 0.451 |

**Abbreviations:** CI, Confidence Interval; HDL-C, high-density lipoprotein cholesterol; IR, insulin resistance; OR, Odds Ratio; PAD, peripheral artery disease; SNPs, single nucleotide polymorphisms; TG, triglyceride. Note: The effect estimates for HDL-C correspond to lower HDL-C levels.

**Supplemental Table S11:** Multivariable Mendelian randomization associations of insulin resistance with CVD risk adjusting for BMI.

| **Exposure** | **SNPs** | **OR** | **95% CI** | **P-value** |
| --- | --- | --- | --- | --- |
| **Hypertension** | | | | |
| IR | 51 | 1.06 | 1.04-1.08 | **1.24E-13** |
| BMI | 143 | 1.06 | 1.05-1.07 | **4.94E-22** |
| **Heart failure** | | | | |
| IR | 49 | 1.22 | 1.04-1.43 | ***0.014*** |
| BMI | 141 | 1.74 | 1.56-1.94 | **1.08E-22** |
| **PAD** | | | | |
| IR | 42 | 1.39 | 0.88-2.21 | 0.163 |
| BMI | 121 | 1.94 | 1.40-2.67 | **5.57E-05** |

**Abbreviations:** BMI, body mass index; CI, Confidence Interval; IR, insulin resistance; OR, Odds Ratio; PAD, peripheral artery disease; SNPs, single nucleotide polymorphisms.
